# ALKBH overexpression in head and neck cancer: potential target for novel anticancer therapy

**DOI:** 10.1038/s41598-019-49550-x

**Published:** 2019-09-13

**Authors:** Tomaš Pilžys, Michał Marcinkowski, Wojciech Kukwa, Damian Garbicz, Małgorzata Dylewska, Karolina Ferenc, Adam Mieczkowski, Andrzej Kukwa, Ewa Migacz, Dominika Wołosz, Damian Mielecki, Arne Klungland, Jan Piwowarski, Jarosław Poznański, Elżbieta Grzesiuk

**Affiliations:** 1 Institute of Biochemistry and Biophysics, Polish Academy of Sciences, Warsaw, Poland; 2 Department of Otolaryngology, Medical University of Warsaw, Warsaw, Poland; 3 Veterinary Research Centre and Center for Biomedical Research, Department of Large Animal Diseases with the Clinic, Faculty of Veterinary Medicine, Warsaw University of Life Sciences, Warsaw, Poland; 4 Department of Pathology, Medical University of Warsaw, Warsaw, Poland; 5 Department of Microbiology, Oslo University Hospital, Oslo, Norway

**Keywords:** Cancer metabolism, Drug development, Head and neck cancer, Proteomics, Predictive markers

## Abstract

The nine identified human homologues of *E*. *coli* AlkB 2-oxoglutarate (2OG) and Fe(II)-dependent dioxygenase, ALKBH1-8 and FTO, display different substrate specificities and diverse biological functions. Here we discovered the combined overexpression of members of the ALKBH family in head and neck squamous cell carcinomas (HNSCC). We found direct correlation of ALKBH3 and FTO expression with primary HNSCC tumor size. We observed unidentified thus far cytoplasmic localization of ALKBH2 and 5 in HNSCC, suggesting abnormal role(s) of ALKBH proteins in cancer. Further, high expression of ALKBHs was observed not only in HNSCC, but also in several cancerous cell lines and silencing ALKBH expression in HeLa cancer cells resulted in dramatically decreased survival. Considering the discovered impact of high expression of ALKBH proteins on HNSCC development, we screened for ALKBH blockers among newly synthetized anthraquinone derivatives and demonstrated their potential to support standard anticancer therapy.

## Introduction

Head and neck squamous cell carcinomas (HNSCC) include a variety of tumours of different epidemiology and ethology, originating in the oral cavity, hypopharynx, nasopharynx, oropharynx, or larynx. HNSCC is the seventh most common malignancy worldwide, responsible for approximately 400 000 deaths per year^1^ In cancer tissues increased dynamic of metabolic processes result in high level of nucleic acid modifications and in consequence, induction of DNA repair systems^2,3^. These system expression leads to the removal of DNA lesions before they become toxic to the rapidly dividing cancer cell, ensuring tumour welfare and creating a major mechanism of resistance to anticancer therapy^3^.

In *Escherichia coli*, AlkB (EcAlkB) protein is a prototypical, one-protein DNA/RNA repair system, part of the so-called “adaptive response”. It is the best known member of the 2-oxoglutarate (2OG) and Fe(II)-dependent dioxygenase superfamily^4^. The human genome encodes nine EcAlkB homologs: ALKBH 1-8 and FTO (fat mass and obesity associated protein)^5^. They fulfil a variety of biological functions, such as DNA repair, involvement in the metabolism of different RNA species, histone demethylation, acting as methyltransferases or taking a part in fatty acid metabolism^6^.

Overexpression of individual ALKBH proteins has been detected in overwhelming majority of various types of cancer^7^, suggesting a pro-carcinogenic role of these proteins (Table 1), e.g. FTO overexpression correlates with increased cancer cell survival in breast cancer, under the conditions of glutamine deficiency^8^. Likewise, *Alkbh2* knockdown in bladder cancer tissue limited tumour development, while ALKBH2 down-regulation sensitized cells to alkylating agents in glioma^9,10^ and cisplatin in lung cancer^11^. Considering these findings, inhibitors of ALKBH proteins could act as anticancer substances. The group of anthraquinones, including rhein and emodin, is particularly promising because these compounds exhibit anti-inflammatory, anti-bacterial, and anti-cancer properties^12^. Moreover, rhein has been shown to inhibit EcAlkB, ALKBH 2, 3, and FTO activity^13,14^.

**Table 1:** Expression of ALKBHs in individual type of cancer.

Cancer	PROTEIN					Citation
	ALKBH2	ALKBH3	ALKBH5	ALKBH8	FTO	
Prostate		**↑**				^40^
Renal		**↑**				^41^
Bladder	**↑**	**↑**		**↑**		^10, 42, 43^
Rectal		**↑**				^44^
Lung		**↑**				^45^
Gastric	**↓**				**↑**	^46, 47^
Pancreas		**↑**				^48^
Endometrial					**↑**	^49^
Breast			**↑**		**↑/↓**	^7, 50, 51^
Glioblastoma	**↑**		**↑**			^52, 53^
Acute myeloid leukemia					**↑**	^21^
Cervical					**↑**	^54^
Hepatocellular		**↑**				^55^

For the first time, we detected overexpression of a majority of the ALKBH proteins in clinical HNSCC samples and high levels of expression in several cancer cell lines. Importantly, some of the proteins changed localization in cancer cells, indicating specific function in different cell compartments, and correlated with clinical tumour parameters. Finally, we modified emodin to improve its inhibitory effect against ALKBHs and show anticancer activity of some of the new derivatives obtained on different cancer cell lines.

## Results

### **Expression of ALKBH proteins correlates with HNSCC development**

#### *ALKBH levels are increased in HNSCC*

The clinicopathological characteristics of the 41 patients are shown in Table 2. The table includes main data concerning patients, TNM tumour parameters, and other diseases.

**Table 2:** Clinicopathological features of HNSCC patients included in this study (n = 41).

**Characteristic N** (**%**) **or mean [IQR]**							
Age		59 [53.5-68.5]		**T classification**			
Weight		73 [58.5-82]		Unknown		10 (24%)	
BMI		25 [20.5-27.9]		1		1 (2%)	
**Sex**				2		12 (29%)	
Male		29 (57%)		3		13 (32%)	
Female		12 (24%)		4		5 (12%)	
Unknown		10 (20%)		**N classification**			
**Tumor location**				Unknown		8 (20%)	
Neck		5 (12%)		0		18 (44%)	
Larynx		29 (71%)		1		4 (10%)	
Tongue		3 (7%)		2		10 (24%)	
Other		4 (10%)		3		1 (2%)	
**Grading**				**M classification**			
Unknown		7 (17%)		Unknown		10 (24%)	
1		1 (2%)		0		31 (76%)	
2		25 (61%)		**TNM stage**			
3		8 (20%)		Unknown		8 (20%)	
				1		1 (2%)	
				2		5 (12%)	
				3		12 (29%)	
				4		15 (37%)	
**Other diseases N** (**%**)							
**Jaundice**		**Asthma**		**Hypertension**		**Problems with blood coagulation**	
Yes	1 (2%)	Yes	2 (5%)	Yes	5 (12%)	Yes	0 (0%)
No	20 (49%)	No	20 (49%)	No	17 (41%)	No	22 (54%)
Unknown	20 (49%)	Unknown	19 (46%)	Unknown	19 (46%)	Unknown	19 (46%)
**Internal organs diseases**		**Peptic ulcers**		**Diabetes**		**Viral hepatitis**	
Yes	1 (2%)	Yes	1 (2%)	Yes	3 (7%)	Yes	1 (2%)
No	20 (49%)	No	21 (51%)	No	18 (44%)	No	22 (54%)
Unknown	20 (49%)	Unknown	19 (46%)	Unknown	20 (49%)	Unknown	18 (44%)

**Figure 1:**
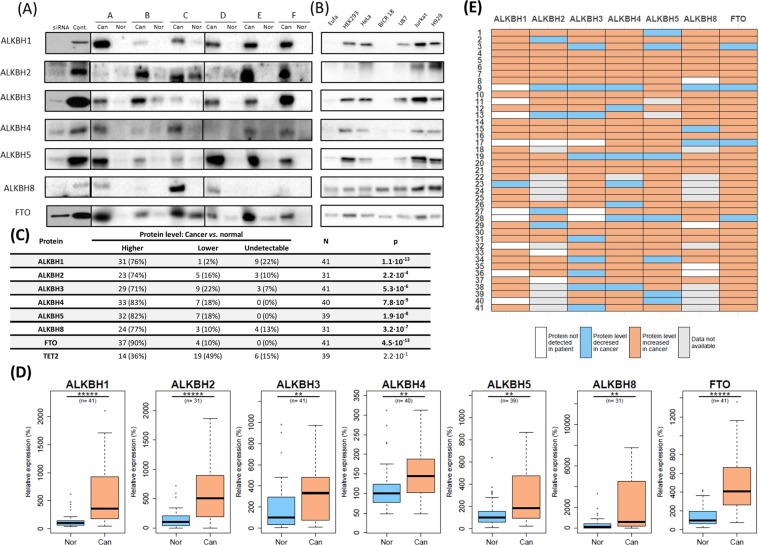
ALKBH expression in HNSCC and indicated cell lines: (**A**) WB analysis of ALKBH expression in HNSCC samples. siRNA - HeLa cells treated with siRNAs directed towards particular ALKBHs; Cont. - HeLa cells not treated by siRNA; Nor - normal periphery; Can- cancer; A-F - tumour samples. (**B**) WB analysis of ALKBH expression in various cell lines: normal, cancer and embryonic. (**C**) ALKBHs expression in cancer and normal tissues from HNSCC patients. Samples were classified into three groups according to the expression level of each protein: (i) stronger signal from cancer than normal surrounding; (ii) weaker signal from cancer than normal surrounding; (iii) no detectable expression of the proteins in the normal and cancer tissue. N – number of patients; p - p-value obtained from the Wilcoxon signed-rank test for paired samples. (**D**) Nonparametric Wilcoxon rank-sum test (for groups) were performed. n- number of samples from each group; P-values with Benjamini-Hochberg adjustment: *p < 0.025; **p < 0.005; ***p < 0.0005; ****p < 0.00005; *****p < 0.000005. (**E**) Heat map of changes of individual protein expression in HNSCC. Fold changes were calculated for tumour *vs*. adjacent normal tissue. White - no detectable expression of particular protein in cancer and adjacent tissue. Blue - decreased relative protein level. Orange - increased relative protein level. Grey - no data gathered.

Using western blot (WB) analysis, we detected overexpression of seven out of the nine ALKBH proteins (ALKBH1, 2, 3, 4, 5, 8, and FTO) in HNSCC cancer tissues, as compared to the surrounding, unaffected tissue (Figs 1A and S1–7). The expression of ALKBH 6 and 7 was not detected by our WB method. We used TET2 dioxygenase expression as the negative control and observed that it was expressed at equal levels in cancer and normal tissue (data not shown). The highest expression levels were observed for ALKBH4, 5, and FTO (Fig. 1C). Further analysis indicated that, among overexpressed ALKBH proteins, the greatest difference between tumour and surrounding tissue was observed for ALKBH2 (5-fold), FTO (4-fold), ALKBH1 (3-fold), and 5 (2-fold) (Fig. 1D). Thus, simultaneous overexpression of the indicated dioxygenases in cancer tissue may be used in cancer diagnosis as a meta-marker. Towards this aim, the best candidates are ALKBH1 and FTO, according to receiver operating characteristic (ROC), where false positive rate were below 26% (Fig. S8).

Analysing the levels of ALKBHs in HNSCC tumours, we observed the simultaneous increase in expression of at least four ALKBH proteins in approx. 88% of patients and at least five ALKBHs in over 50% of patients. More than 25% of patient samples tested showed high expression of all seven ALKBH proteins (Fig. 1E). This implicates similar substrate specificity and/or common regulation of these proteins. To find out if ALKBH overexpression is a common phenomenon in cancer cells, we also assessed expression of ALKBH proteins in various cancerous cell lines: HeLa (cervix), U87 (brain), BICR 18 (larynx), Jurkat E6-1 (peripheral blood) and H929 (bone marrow), and embryonic HEK293 (kidney). As a model of normal tissue we used EUFA30 (fibroblast) (Fig. 1B). In almost all of the cancerous cell lines—despite different origin—we observed expression of all of the ALKBH proteins tested. The highest expression of ALKBH proteins was detected in HeLa, Jurkat, H929, and HEK293, while the lowest was detected in BICR18 and EUFA30, the control.

### *Correlation between the levels of expression of ALKBH proteins*

**Figure 2:**
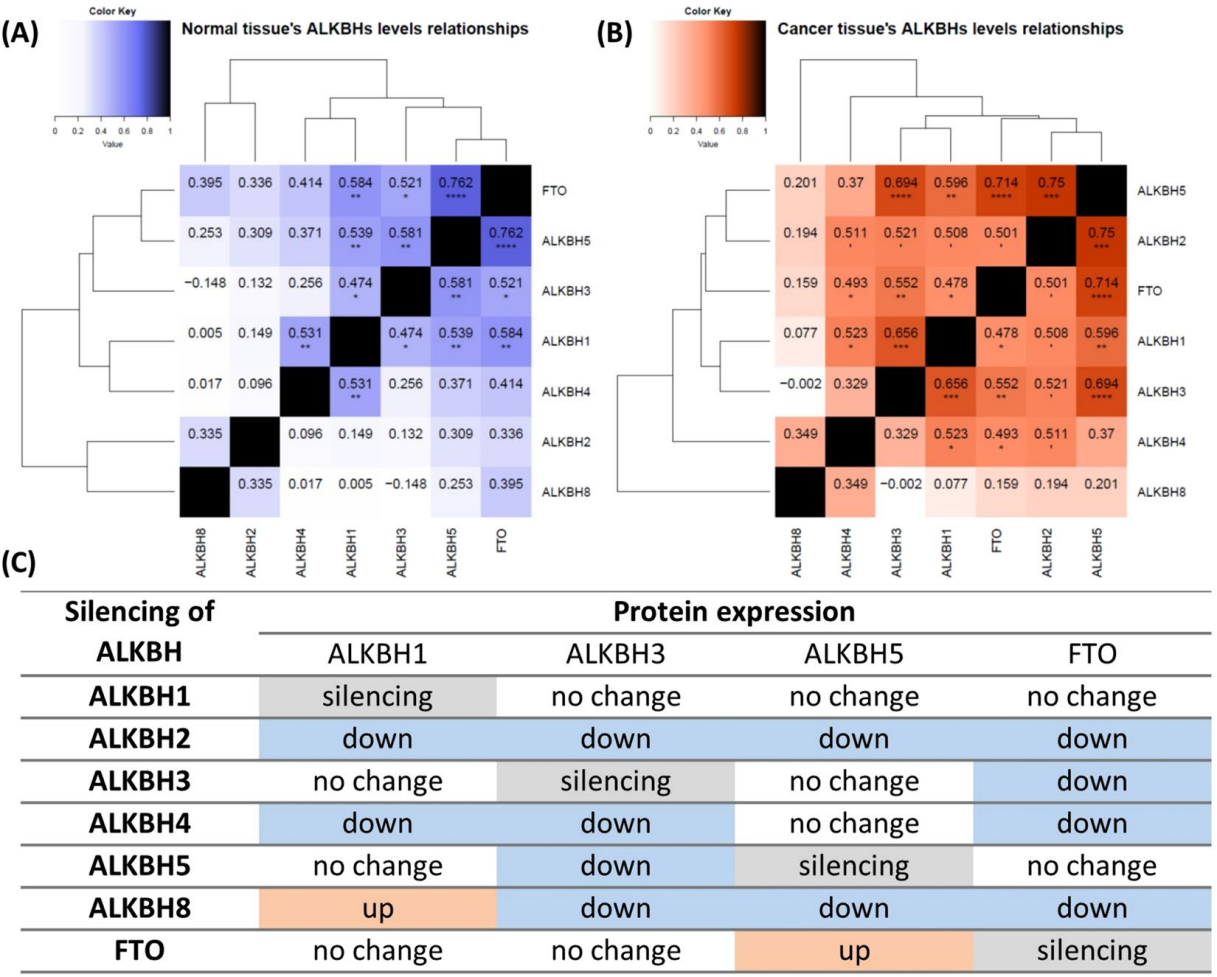
Relationships between protein levels within ALKBH family members. (**A**) Relationship between the levels of ALKBH proteins in healthy surrounding tissue and (**B**) HNSCC tumour. Each tabulated entry presents the Spearman’s rank correlation coefficient and indicates the p-value of correlation. Hierarchical cluster analysis of matrices presents similarities between examined ALKBHs. P-values with Benjamini-Hochberg adjustment: ‘p < 0.005; *p < 0.0025; **p < 0.0005; ***p < 0.00005; ****p < 0.000005. (**C**) Impact of individual ALKBH protein silencing on expression of its homologs in HeLa. Silencing was done with the use of small interfering RNA, ALKBH protein levels were measured by WB.

Next, we assessed the relationship between the expression levels of individual ALKBHs protein in normal surrounding tissue and in HNSCC (Fig. 2A,B). In the healthy periphery, we identified three correlated pairs, with the highest Spearman’s rank correlation coefficients (ρ > 0.55, p < 10^−3^) calculated for ALKBH5-FTO, ALKBH1-FTO, and ALKBH3-ALKBH5 pairs. In the cancer tissue, we observed a similar correlation involving ALKBH5-FTO, ALKBH3-ALKBH5, and ALKBH1-ALKBH5, but also noted that the level of ALKBH2 was correlated with that of ALKBH5 and ALKBH1 with ALKBH3.

To investigate further these correlations, we used small interfering RNA (siRNA) for *ALKBH1*, *2*, *3*, *4*, *5*, *FTO* genes and using WB analysis, we recognized the expression levels of ALKBH1, 3, 5 and FTO (Fig. 2C). The HeLa cells were chosen as highly expressing the ALKBH proteins. In the case of *ALKBH2*, *4*, and *8* genes, their silencing led to the downregulation of almost all ALKBHs tested. Silencing of *ALKBH1* or *FTO* did not influence the level of the investigated proteins. Interestingly, only in two cases we observed the opposite effect, namely, *ALKBH8* silencing led to the elevated level of ALKBH1, while *FTO* silencing increased ALKBH5 level. Moreover, we noticed downregulation of ALKBH3 and FTO after the silencing of *ALKBHs* in the most cases.

### *ALKBH3andFTOlevels are related to the clinical tumour parameters*

**Figure 3:**
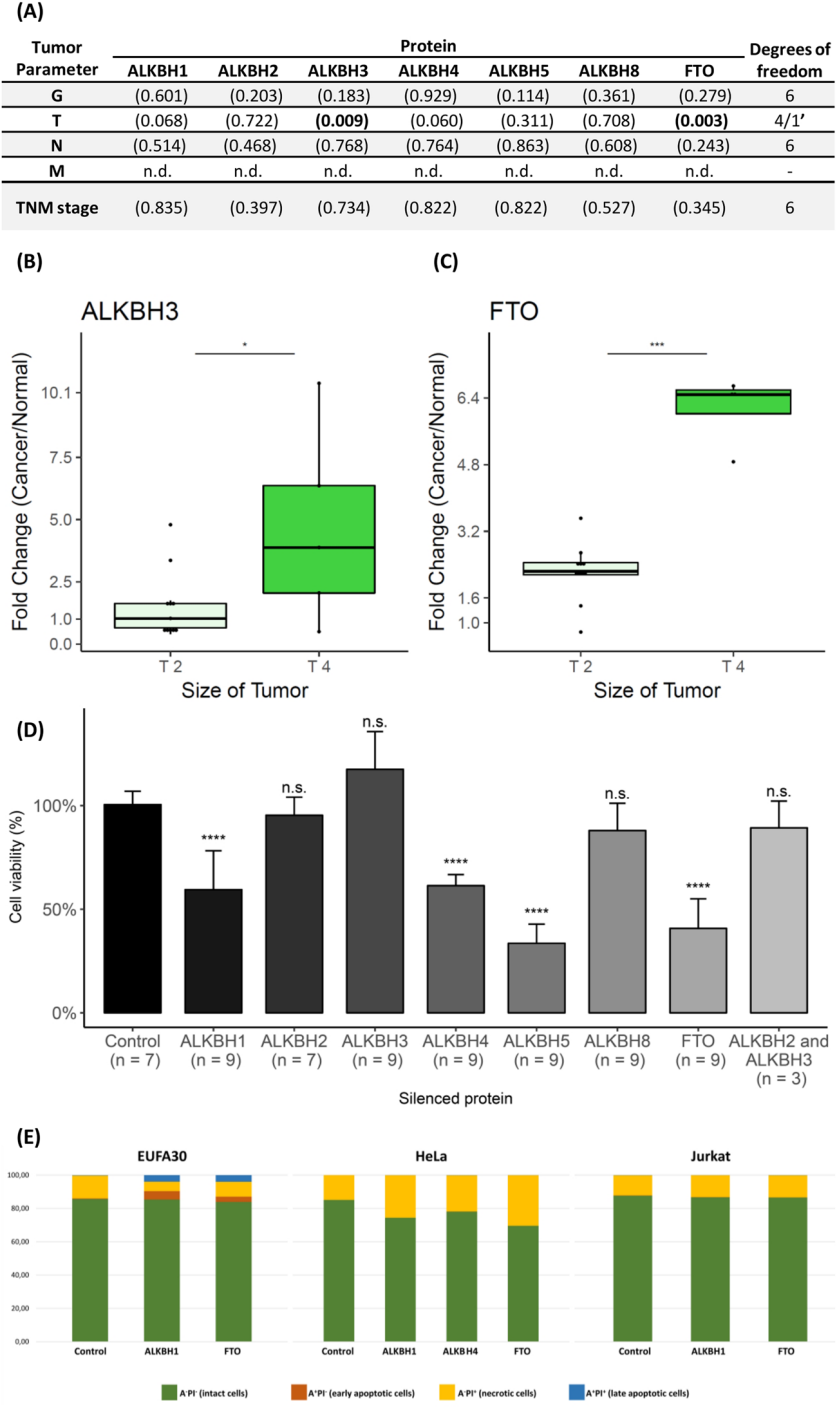
Relationship between ALKBH proteins level and tumour parameters and cancer cell viability. (**A**) Analysis of variance (ANOVA) of ALKBH protein levels in HNSCC tumours. Each tabulated entry shows F-value and p-value of F-test, providing information on the effect of the level of the particular ALKBH protein on the specific tumour parameter: G – tumour invasiveness, T, N, M - The Union for International Cancer Control (UICC) parameters. The sample size for ALKBH2 and ALKBH8 was too small to execute ANOVA with other ALKBH proteins, a separate analysis was performed for these two proteins. (**B**,**C**) Relationship between selected ALKBH protein and tumour size. P-values with Benjamini-Hochberg adjustment: *p < 0.05, ***p < 0.001. (**D**) Survival assay of HeLa cells treated with siRNA for a given ALKBH protein. HeLa cell viability was assessed 48 h after treatment. P-values with Benjamini-Hochberg adjustment: ****p < 0.0001. (**E**) Flow cytometry analysis of EUFA30, HeLa, and Jurkat cells stained with Annexin-FITC and propidium iodide. Cells were treated with 34 nM of siRNA on *ALKBH1*, *4*, or *FTO* for 48 h.

In order to assess the relationship between ALKBH level and cancer progression, we compared protein expression levels with tumour parameters. We detected significantly higher levels of ALKBH3 and FTO in larger primary tumours (type T4 in TNM classification), as compared to smaller ones (type T2 in TNM classification) (Fig. 3A). Interestingly, in type T2 tumours, the level of ALKBH3 was similar or slightly higher than in adjacent tissue, whereas in type T4 tumours ALKBH3 level was strongly elevated in comparison to unaffected surrounding tissue. Meanwhile, increased levels of FTO were observed in all examined groups (Fig. 3C). Thus, the content of ALKBH3 and FTO increases along with the tumour size. We did not observe any other correlations between tumour size or primary tumour extension and the other ALKBH proteins; ALKBH levels were not correlated with tumour invasiveness (G parameter), metastasis (N parameter), or TNM stage (Fig. 3A). Finally, we assessed the impact of ALKBH expression level on HeLa viability. Using siRNA, we silenced single/double ALKBH proteins and found that silencing ALKBH1, 4, 5, and FTO significantly influenced cell viability (Fig. 3D). On the other hand, silencing ALKBH2, 3, 8 and the combined silencing of ALKBH2 and 3 did not influence cell viability.

When silencing performed in EUFA30, HeLa, and Jurkat cell lines with siRNAs targeted towards selected *ALKBHs*, we observed that downregulation of ALKBH1, 4, or FTO slightly promoted necrosis in HeLa cells to the values of 25.5, 21.7, and 30.6%, respectively (Fig. 3E). On the other hand, Jurkat cells did not show any sensitivity to siRNAs used. Although in EUFA30 cells, ALKBH1 and FTO siRNAs induced apoptosis (9 and 7%, respectively), the levels of cell viability were still in the range of the control.

### Elevated level of RNA N^6^-methyladenosine in HNSCC

**Figure 4:**
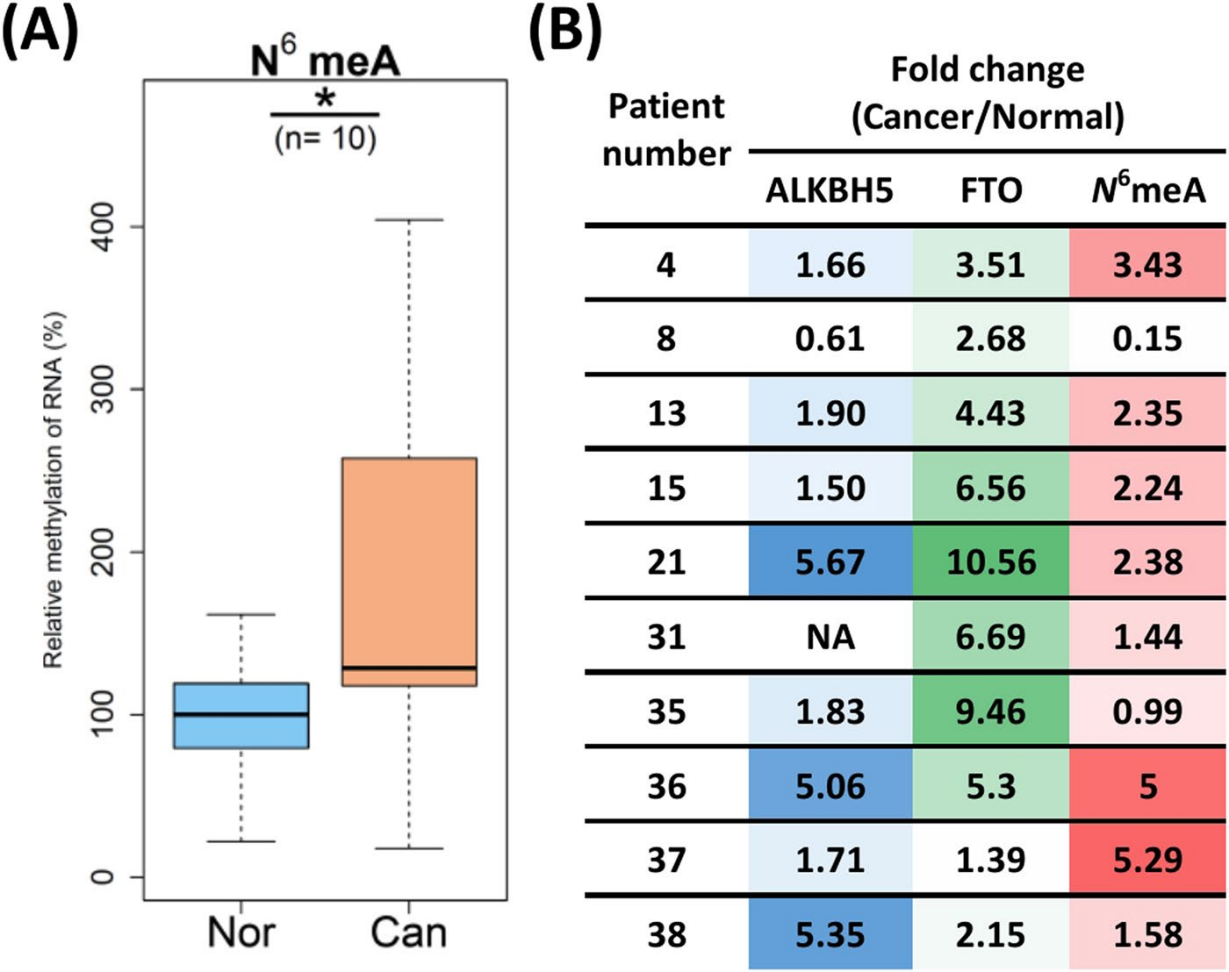
The level of *N*^6^meA in cancer and normal tissue. (**A**) For pairs, nonparametric Wilcoxon signed-tank test was performed. n - paired number; *p < 0.05; Nor- normal periphery; Can- cancer. (**B**) Comparison of relative expression levels of ALKBH5, FTO and *N*^6^meA. Deeper colours represent higher level of ALKBH5 (blue), FTO (green) or *N*^6^meA (red).

Having discovered high level of ALKBH proteins in HNSCC, we decided to assess the level of *N*^6^-methyl-adenosine (*N*^6^meA), the most abundant RNA modification in eukaryotic cells and the substrate for FTO and ALKBH5 proteins. Surprisingly, despite high expression of FTO/ALKBH5 in cancer tissue, the level of *N*^6^meA was also significantly elevated, in comparison to normal tissue (Fig. 4A). Nevertheless, we observed a great deal of variation in the FTO/ALKBH5 *vs N*^6^meA ratio in individual patients (Fig. 4B).

### The cellular localization of ALKBHs in HNSCC cells

**Figure 5:**
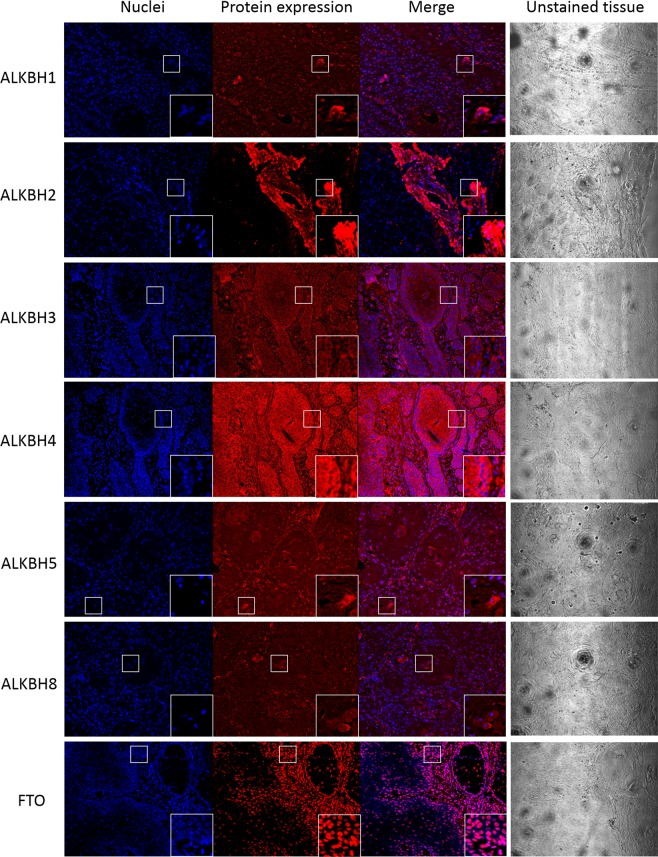
ALKBH proteins expression in the HNSCC tissue in II stage accordingly to TMN stale visualized using confocal microscopy. Cell nuclei staining by Hoechst 3558 visualized as blue fluorescence (nuklei panel), ALKBH proteins expression was visualized using specific primary antibodies and secondary antibody conjugate with AlexaFluor 568 as red fluorescence (protein expression panel), merge (merge panel), transparent view of unstained tissue (unstained protein panel). Objective 20x. Additional zoom (2.5x) of part of view marked by white frame was performed to visualized cellular localization of ALKBH proteins in single cell. Basic microscope settings were as follows: scan layer = 200 μm; kalman = 8; offset = 0%, scanning: sequential for each channel: for Alexa Fluor 568 - green laser (HeNe 543 nm); for Hoechst 3558 (UV Ar, 351 nm).

Confocal microscopy analysis was used to examine any changes in the subcellular localization of ALKBH proteins in HNSCC cells, as compared to the normal, surrounding tissue (Table S1). For ALKBH1, 4, 8, and FTO we observed the expected, previously-described localization. However, for ALKBH2 and 5, we found that these nuclear proteins were also highly expressed in cytoplasm. Additionally, we observed that ALKBH3 was expressed only in cytoplasm (Fig. 5). These changes in protein localization suggest that ALKBHs may play different roles in cancer and normal cell metabolism.

## Design of ALKBH Inhibitors

The western blot analysis and immunofluorescence on clinical HNSCC samples and RNA interference in human cell line indicate the involvement of ALKBH proteins in cancer survival and growth. As a result, the ALKBH inhibitors may offer a new approach to anticancer therapy. Towards this aim, we performed molecular modelling of ALKBH proteins with a set of selected antraquinones with proven anti-cancer activity, as well as newly synthetized chloro-derivatives.

### *In silicomodelling of the ALKBH - anthraquinone (enzyme-ligand) complexes*

**Figure 6:**
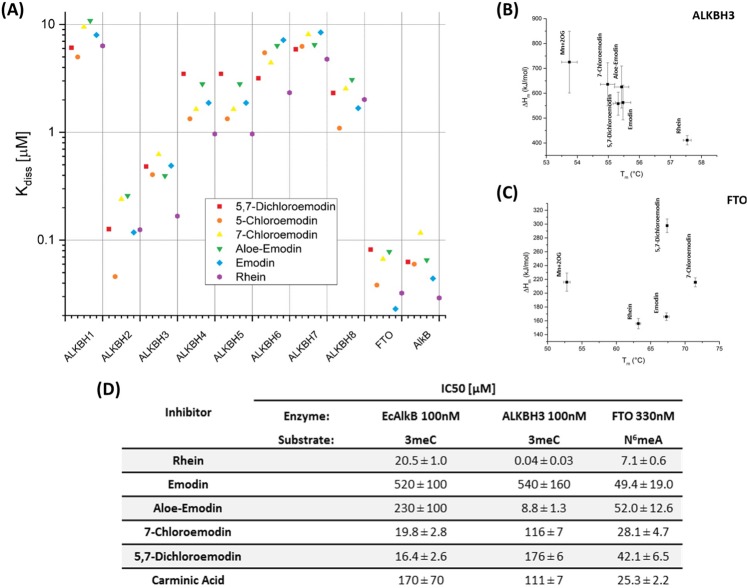
Design and *in vitro* examination of ALKBHs inhibitors. (**A**) Results of *in silico* screening for the binding of anthraquinone derivatives by ALKBH proteins. Molecular docking indicated that FTO and EcAlkB are the best targets, followed by ALKBH2 and ALKBH3, albeit each of these proteins displayed specificity towards particular ligands. Other ALKBH proteins virtually did not bind any of the ligands tested. (**B**,**C**) The results of the thermal shift assay for ALKBH3 and FTO in the presence of tested anthraquinones. ΔH - enthalpy, T_m_ - protein melting temperature. (**D**) Inhibitory effect of anthraquinones on EcAlkB protein and its human homologs.

Because no structures of the proper forms of holoenzyme complexes with anthraquinone derivatives are accessible in PDB (Fig. S9), we performed *in silico* screening for all ALKBH proteins by means of homology modelling, followed by molecular docking. The binding affinities estimated for 60 various complexes of the ten ALKBH proteins (ALKBH1-8, FTO, and EcAlkB) with six anthraquinone derivatives ranged from 0.02 to 10 μM (Fig. 6A). EcAlkB and FTO were the most preferred targets for all ligands, ALKBH2 and ALKBH3 were clearly less preferred, while the remaining six proteins did not bind the tested anthraquinones (K_diss_ > 1 μM). These results are in agreement with previous experimental data demonstrating that rhein inhibits bacterial EcAlkB and eukaryotic ALKBH 2, 3, and FTO proteins^13,14^.

The structure of the FTO-rhein complex was close to that accessible in PDB, but the ligand was placed deeper at the binding site (RMSD < 2 Å). In contrast to ALKBH3—for which the carboxyl group of rhein interacts with Arg118 and Arg131 (Fig. S10) — no salt-bridges were identified for FTO-ligand interactions; thus, not surprisingly, 7-chloroemodin was found to be the best ligand, whereas emodin and 5-chloroemodin scored slightly lower (Figs S11 and S12).

### *Impact of anthraquinones on stability and enzymatic activity of AlkB, ALKBH3, and FTO*

We tested the affinity of our newly synthesized anthraquinones, 7-chloroemodin and 5,7-dichloroemodin, (Fig. S13) for ALKBH3 and FTO and assessed their effect on the enzymatic activity of targeted proteins using the thermal shift assay (TSA). The thermodynamic parameters for thermal denaturation of both proteins were measured for their *holo* forms, in the presence of 2OG and Mn^2+^, an analogue of Fe^2+^.

The strongest ligand for ALKBH3 was rhein (Figs 6B and S14A), because its addition increased the melting temperature the most. Emodin, aloe-emodin, and 5,7-dichloroemodin bound to ALKBH3 slightly more weakly.

All of the tested ligands increased the melting temperature of the FTO *holo* form (52.8 ± 0.6 °C) by more than 10 °C (Fig. 6C). The most pronounced effect was observed for 7-chloroemodin and 5,7-dichloroemodin, with these complexes melting at 71.5 ± 0.2 °C and 67.4 ± 0.1 °C, respectively.

For EcAlkB, ALKBH3, and FTO, the inhibitory effect was further quantified by measuring inhibition of demethylation activity *in vitro* (Fig. 6D). We used 3-methylcytosine (3meC) on TT(3meC)TT pentamer as a substrate and the anthraquinones listed in Fig. 6D as inhibitors. The best EcAlkB inhibitors were 7-chloroemodin and 5,7-dichloroemodin, with IC_50_ of 19.8 μM and 16.4 μM, respectively, followed by rhein with IC_50_ of 20.5 μM. The remaining anthraquinones displayed at least 10-fold lower activity. Enzymatic activity assay also confirmed that rhein is the best inhibitor of ALKBH3, with IC_50_ of 40 nM (Fig. S14). Aloe-emodin displayed lower activity (IC_50_ 8.8 μM) and the other anthraquinones were inactive (IC_50_ > 100 μM). For the case of FTO, activity that was monitored using *N*^6^meA demethylation assay and rhein was the most efficient inhibitor with IC_50_ of 7.1 μM, followed by 7-chloroemodin with IC_50_ of 28.1 μM, which was more active than emodin. In summary, rhein, 7-chloroemodin, and 5,7-dichloroemodin are the most effective ALKBH inhibitors, but possess different affinities towards the individual proteins.

### *Cytotoxic effect of anthraquinones on normal and cancer cells*

**Figure 7:**
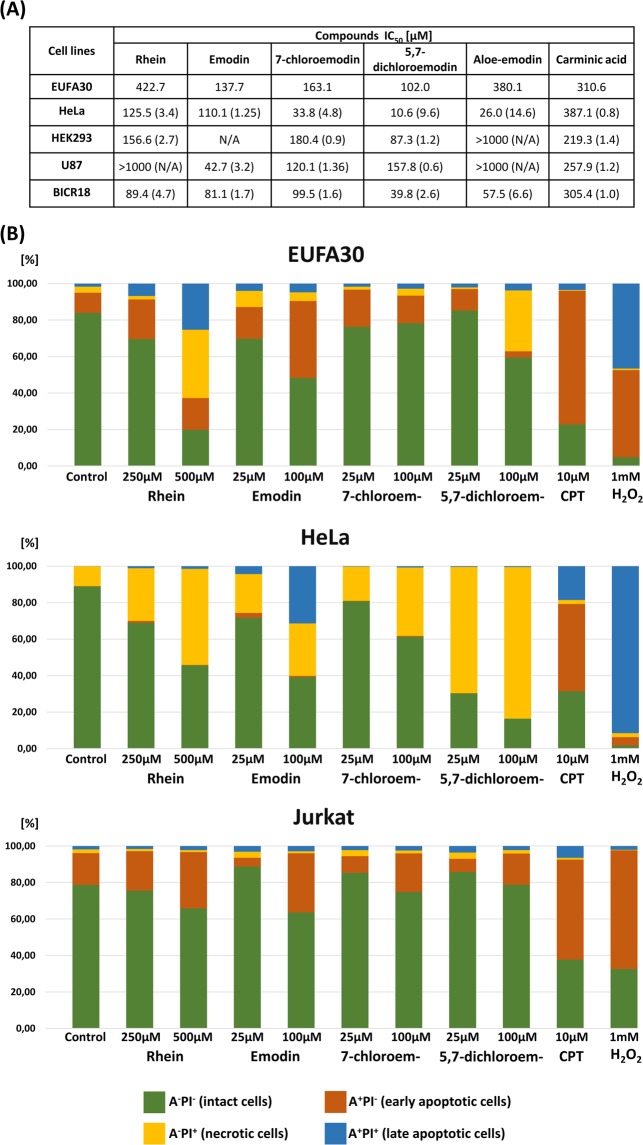
Viability assay and flow cytometry analysis for indicated anthraquinones. (**A**) Cytotoxic effect of natural anthraquinones and newly synthetized derivatives on EUFA30, HeLa, HEK293, U87, BICR18 cells after 48 h of treatment. Table includes IC_50_ value (μM). Therapeutic index is provided in brackets (TI = IC50 normal cells/IC50 cancer cells). (**B**) Flow cytometry analysis of EUFA30, HeLa, and Jurkat cells stained with Annexin-FITC and propidium iodide. Cells were treated with 250 or 500 µM rhein or 25 µM and 100 µM emodin, 7-chloroemodin and 5,7-dichloroemodin for 48 h. CPT - camptothecin (10 µM) and H_2_O_2_ (1 mM) were used as positive controls.

We evaluated the cytotoxic efficacy of the compounds under study, on cancerous HeLa, U87, BICR18, embryonic HEK293, and normal EUFA30 cells (Fig. 7A). We found that emodin is the most cytotoxic among all natural anthraquinones tested. Moreover, it is more toxic to cancerous than healthy cells. The IC_50_ values for emodin were 110.1, 42.7, and 81.1 μM for HeLa, U87, and BIRC18, respectively. Its therapeutic index (TI) varied from 1.25 to 3.2. Within newly synthetized compounds, 5,7-dichloroemodin was the most potent, with IC_50_ equal to 10.6 and 39.8 μM and a therapeutic indexes of 9.6 and 2.6 for HeLa and BICR18, respectively. Summarizing, emodin and 5,7-dichloroemodin showed higher cytotoxicity against cancerous than healthy cell lines. Noteworthy, the biggest therapeutic indices, equal 14.6 μM, showed aloe-emodin against HeLa cells. Unfortunately, this compound was not cytotoxic against HEK293 and U87 cell lines. Additionally, HeLa cells were less resistant to anthraquinones.

Apoptosis induction after rhein, emodin, 7-chloremodin, and 5,7-dichloroemodin treatment was assessed using flow cytometry in normal (EUFA30) and cancerous cell lines (HeLa, Jurkat) (Fig. 7B). EUFA30 cells were more sensitive to natural anthraquinones, rhein and emodin, than to the newly synthetized derivatives: after treatment with 500 μM rhein, early apoptosis stage increased approximately 2-fold, late apoptosis 15-fold, and necrosis increased 11-fold, compared to untreated control (Fig. 7B). HeLa cells were much more sensitive to 7-chloroemodin and 5,7-dichloroemodin than EUFA30. The number of necrotic HeLa cells increased to 69.2% and 83.1%, in the presence of 25 μM and 100 μM 5,7-dichloroemodin, respectively. For 7-chloroemodin, the respective values were 18.8% (for 25 μM) and 37.6% (for 100 μM), as compared to 10.8% in the case of untreated control. Emodin showed similar effect as anthraquinone derivatives, but also promoted late apoptosis. Overall, Jurkat cells were the least sensitive. Interestingly, anthraquinones cause mostly apoptosis in EUFA30 and Jurkat cell lines, while necrosis prevails in HeLa.

In summary, the natural anthraquinone emodin and our novel derivative 5,7-dichloroemodin demonstrate promising potential as inhibitors for anticancer therapy.

## Discussion

In the last decade, the family of AlkB dioxygenases has been extensively studied, especially in the context of new substrates, but also in terms of their role in the development of so-called “diseases of affluence”, including obesity, type-2 diabetes, cancer etc. Several recent publications have reported increased levels of individual ALKBHs in various types of human cancer (Table 1). Here we demonstrated that in HNSCC, the expression level of seven out of nine ALKBHs (1, 2, 3, 4, 5, 8, and FTO) was significantly increased in 68-90% of the samples. This shows, for the first time, that not just one single ALKBH, but the whole group of ALKBH proteins is overexpressed in HNSCC tumours. At least four different ALKBH proteins were highly expressed in 88% of tested tumours and in 25% of cases, all seven proteins were overexpressed. We found highly expressed ALKBH proteins in several cancerous cell lines, suggesting that the simultaneous overexpression of several members of ALKBH family proteins is a common process accompanying cancerogenesis. This hypothesis is supported by the observation of strong co-expression of particular ALKBH pairs in HNSCC, namely ALKBH1 with 3 and ALKBH2 with 5. Moreover, in the case of HeLa cells, the silencing of *ALKBH2*, *4*, or *8* genes reduced expression of other homologs (Fig. 2C). Keeping in mind that FTO and ALKBH5 share the same substrate specificity^15,16^, not surprisingly that the silencing of the *FTO* gene leads to upregulation of ALKBH5 protein. Furthermore, one can suppose that the ALKBHs co-express in the similar way and can be co-regulated at the expression level. Further, silencing of ALKBH1, 4, 5, and FTO markedly decreased HeLa cancer cell survival (Fig. 3), suggesting involvement of these ALKBHs in cancer development by DNA/RNA/protein modification. In contrast, silencing of* ALKBH2, 3, 8* or both, *ALKBH2* and *3*, did not affect cancer cell viability. This is in agreement with prior reports showing that knocking down *ALKBH2* or *3* did not influence mice phenotype and viability^17^. We can conclude that overexpression/co-expression of the whole group of ALKBH proteins correlates with cancer development, including its different types, indicated by: (i) the high expression of almost all of the ALKBHs in HNSCC and in various cancer cell lines; (ii) decreased cancer cell viability following ALKBHs silencing; (iii) previously reported high expression of single ALKBHs in different types of cancer; (iv) the siRNA-treated mice with developed cancer show diminishing tendency of cancerous tissue. Summing up, overexpression of a single or a group of ALKBH proteins could be used as a marker for cancer diagnosis^18^. The samples obtained by biopsy (or surgery) could be subjected to Western blot analysis and adequate chemotherapy, e.g. combination of alkylating agents and antraquinone derivatives, could be established.

Further, we demonstrated that ALKBH3 and FTO levels are correlated with primary tumour size (T in TNM classification): protein expression was higher in larger T4 tumours, as compared to smaller T2 tumours. The correlation between increased FTO expression and tumour size may suggest a new regulatory role for this protein in tumour development. On the other hand, the high amount of the repair protein ALKBH3 present in larger tumours may be explained by accumulation of alkylation damage to DNA/RNA, due to metabolic instability of the developed cancer. We can presume that other ALKBH proteins may also promote or sustain tumour welfare, considering their high expression levels.

Interestingly, we also observed an increased global level of *N*^6^meA in HNSCC, the most abundant internal modification in mammalian mRNA, suggesting an abnormal methylation profile in HNSCC. *N*^6^meA is involved in mRNA stability, translation efficiency, splicing, RNA-protein interactions, and more^15^. It is also responsible for cancer stem cell pluripotency, cell differentiation, proliferation and metastasis^19^. Our findings reflect a discrepancy in FTO/ALKBH5 action as the demethylase regulating *N*^6^meA level in mRNA in normal *vs* cancer cells. These findings are supported by the results of Merkeinestein group who has shown that an additional copy of FTO gene did not influence the global level of *N*^6^meA^20^. However, FTO may impact demethylation of selected transcripts, like in acute myeloid leukaemia where FTO plays a critical oncogenic role through demethylation of ASB2 and RARA mRNA (protein that are responsible for blood cell differentiation)^21^, also demethylation of RUNX1T1 (negative regulator of adicipogenesis)^15^, C/EBPβ (stimulator of hepatocytes and adipocytes growth)^22^, PPARγ (adipocytes differentiation)^23^, GAP-43 (growth of axons)^24^. Collectively, these findings prove that FTO is involved in various cancer development.

The different role of ALKBHs in normal *vs* cancer cells was also confirmed by the results of subcellular localization using immunofluorescence. In HNSCC we observed the unexpected localization of nuclear proteins ALKBH2 and 5 in the cytoplasm, while ALKBH3 was found only in cytoplasm. These changes in subcellular localization suggest that these proteins can be involved in new, unknown functions that may be connected with tumour growth. Further, changes in localization of the remaining ALKBH proteins may be the result of a specific pathological state and can be taken under consideration as a prognostic marker for cancer transformation^25^.

The relatively high expression of ALKBHs in tumor tissues correlate with cancer development^26,27^ making these proteins the potential targets for anticancer therapy. We focused on natural antraquinones as inhibitors of ALKBHs and to improve the solubility and the inhibitory effect of these compounds, we substituted hydrogen(s) with chloride(s) atoms in emodin. Molecular docking analysis and *in vitro* activity assay revealed that all tested antraquinones strongly interact with EcAlkB, FTO, ALKBH2, and ALKBH3. Rhein shows the strongest inhibitory effect among compounds under study; however, emodin and newly synthetized 5,7-dichloemodin were the most toxic against cancer cell lines. These data are in agreement with the results published by Li and co-workers (2016), who established the IC_50_ values for rhein against EcAlkB and ALKBH3 were 12.7 and 5.3 μM, respectively^14^. Similarly to our observation, the authors did not found any cytotoxic effect of rhein against U87 cells. Chen and co-workers (2012) calculated the rhein IC_50_ against FTO protein activity in the range of about 30 μM^13^. The similar results were obtained by Wang and co-workers, where emodin has shown higher cytotoxic effect than rhein on human proximal tubular epithelial HK-2 cell line, and only emodin induced apoptosis^28^. On the other hand, the facts that Jurkat cells, although expressing ALKBHs at higher level than HeLa cells, were more resistant to antraquinones, and that EUFA30 cells poorly expressing ALKBHs were least sensitive imply that the emodin derivatives act not only by ALKBHs inhibition but also interfere with other metabolic pathways. The assessment of apoptosis/necrosis in EUFA30, HeLa, and Jurkat cells treated with siRNAs directed towards selected *ALKBHs* confirmed that at least, but not last, the inhibition of the dioxygenases studied leads to cell death, since the only visible impact of siRNAs could be observed in the case of HeLa cells, similarly to the results shown for anthraquinones. If this were not the case, the Jurkat cells would be more sensitive than HeLa. Indeed, it was shown that rhein and emodin also interact with other proteins. Emodin inhibits VEGFR2, PI3K, and p-AKT expression in colon colorectal HCT116 cells^29^. Moreover, emodin inhibits tumor necrosis factor alpha-induced calcification via the NF-kB pathway^30^. Additionally, rhein influences cytoskeleton regulation, protein folding, or transcription control in breast cancer MCF-7 cell lines^31^. Also, the synergistic way of action cannot be excluded.

After the anthraquinone treatment, we observed mainly apoptosis in EUFA30 and Jurkat cells, and necrosis in HeLa cells. Varela-Ramirez and co-workers (2011) obtained similar results testing diphenylmethyltin chloride (DPMT) on various cell lines. They observed that DPMT induces necrosis (more than 40% of cells) on HeLa cells and mostly apoptosis on Kit-225 (human T lymphoma) or EL4 (murine T lymphoblastic)^32^.

For the first time we proved majority of human ALKBH proteins overexpress in HNSCC. Based on the key role of ALKBHs in HNSCC development, ALKBHs should be considered as promising new targets for the diagnosis and treatment of various type of cancer that exhibit high level of ALKBH proteins. In particular, the synthesis and use of new, more powerful ALKBH inhibitors could be a promising strategy supporting anti-cancer therapies based on alkylating agents. This new, modern approach could be used not only in head and neck cancers, but also in other types of cancer that demonstrate ALKBH overexpression.

## Materials and Methods

### Clinical samples

41 tissues (blinded samples) of head and neck carcinomas (29 males and 12 females) were collected at the Department of Otorhinolaryngology, Faculty of Medicine and Dentistry at Medical University of Warsaw between 2011 and 2014. 73.3% of these patients suffered from laryngeal cancer. All of the cancers were histologically verified as squamous cell carcinomas. The research was performed in accordance with the relevant guidelines and regulations. Tumours were classified according to the TNM staging system. Patients were not treated with chemo- or radiotherapy. Samples after histopathological examination were available in archives. Patients agreed to use the samples according to current procedure. Ethical Comity accepted biochemical tests on the samples prior to subsequent utilization according to routine procedures of biohazard. Samples were anonymized with no access to patient personal data. The clinicopathological data are presented in Table 2.

### Tissue samples and western blot analysis

Frozen samples were homogenized in liquid nitrogen and extracted with RIPA buffer (Sigma-Aldrich) supplemented with 50 mM EDTA and 4 mM PMSF in the presence of protease inhibitor cocktail (Sigma-Aldrich). Cellular debris was spun down and supernatant protein content was measured using the Bradford assay (Bio-Rad). Samples were diluted with SDS-PAGE loading buffer to a protein concentration of 3 μg/μl and 10 μl was loaded onto Mini-PROTEAN TGX 4-15% gradient gels (Bio-Rad). The western blot analysis was performed with specific primary monoclonal and polyclonal antibodies used at dilutions: 1:200-500 against ALKBH1, ALKBH3, FTO (Santa Cruz Biotechnology), ALKBH2, ALKBH5, ALKBH6, ALKBH7, ALKBH8 (Sigma-Aldrich) and ALKBH4 (Proteintech) with appropriate 1:2000 secondary anti-mouse IgG antibody (Sigma-Aldrich) or anti-rabbit IgG antibody (Santa Cruz Biotechnology) conjugated with horse-radish peroxidase. All incubations were performed in 5% milk/PBST (PBST – phosphate buffer saline with 0.1% TWEEN-20). Chemiluminescence was measured using the ChemiDoc MP Imaging System (Bio-Rad). Total protein was standardized in four steps: (i) equal masses of the tissue were taken for extraction in RIPA buffer (50 mg); (ii) extract was then assayed by Bradford assay for protein content; (iii) equal amounts were loaded on the gel and verified by Coomassie blue staining; (iv) protein transferred to the nitrocellulose membrane was visualized by Ponceau-S reversible staining prior to the final western blot. Additionally, in the case of several proteins, primary antibodies recognized epitopes of different molecular mass. To define which band corresponds to which examined ALKBH protein, we decreased the protein levels by using RNA interference.

### *N*^6^meA RNA methylation assay

Frozen samples were homogenized in liquid nitrogen. Total RNA was extracted by TRIzol Reagent (ThermoFisher) according to the manufacturer’s protocol. Concentration and purity of RNA was measured spectrophotometrically by Nanodrop 2000c (ThermoScientific). Two hundred ng of total RNA was used in quantification of *N*^6^meA level with use of *N*^6^meA RNA Methylation Quantification Kit (EpiQuik). The assay was carried out according to the manufacturer’s protocol. Absorbance at 450 nm was measured using a scanning multiwall multimode spectrophotometer (DTX 880, Beckman Coulter). To determine relative methylation status of RNA samples, following Eqs. (1) and (2) were used:

1 $$Relative\,{N}^{6}MeA\,of\,sample=\frac{(Sample\,{A}_{450}-NC\,{A}_{450})/S}{(PC\,\,{A}_{450}-NC\,{A}_{450})/P}$$

2 $$Relative\,methylation\,of\,RNA\,in\,sample=\frac{Relative\,{N}^{6}\,MeA}{No{r}_{median}}\ast 100 \% $$

where: Sample A_450_ – Absorbance of a sample, NC A_450_ – Absorbance of a negative control, PC A_450_ – Absorbance of a positive control, S – Amount of RNA in a sample (200 ng), P – Amount of *N*^6^meA in a positive control (0.5 ng), Nor_median_ – median of the relative *N*^6^meA level in a normal adjacent tissues.

### Immunofluorescence analysis

Antigen retrieval in 5 µm rehydrated tissue sections was performed by boiling the slides in citrate buffer. Non-specific binding was blocked with 1% BSA/PBS at RT for 40 min. The same primary antibodies as those used for western blots were used at 1:50-1:200 ratio with 0.1% BSA/PBS by overnight at 4 °C. Secondary antibody (Thermo Fisher Scientific, AlexaFluor 568, A-11011) at a dilution of 1:100-1:400 was incubated at RT for 1 h. Simultaneously, incubation without primary antibodies was performed to exclude false immunofluorescence signal from nonspecific binding of secondary antibodies or from autofluorescence of the tissue. Cell nuclei were stained with Hoest 3558 at 10 μg/ml for 30 s at RT (Life Technologies). Confocal microscopy (Olympus FV500) with Fluoroview v5 software was used to obtain images—imaging details are provided in the figure legend.

### Synthesis of chloroemodins

The 114 mg of emodin (0.42 mmol, 1 eq) was dissolved in 5 ml of tetrahydrofuran (THF) and treated with 169 mg (1.26 mmol, 3 eq) of *N*-chlorosuccinimide. The reaction mixture was stirred in RT for 18 h, then evaporated with silica gel (Silica Gel 60 M (0.040-0.063 mm, E. Merck) and purified by column chromatography using 5% methanol in chloroform for monochloroemodins and 20% methanol in chloroform for 5,7-dichloroemodin^33^. The following yields were obtained: 19.8 mg (15.5%) for 7-chloroemodin and 12.6 mg (8.8%) of 5,7-dichloroemodin.

### Expression and purification of hFTO, AlkB, and ALKBH3 proteins

The plasmid with hFTO was constructed using StarGate cloning system (IBA Life Science) and introduced into *E*.*coli* BL-21. Bacteria were cultured in 37 °C to OD_600_ = 1, induced with 1 mM IPTG, cultured for 16 h at 16 °C, harvested, resuspended in PBS buffer with protein inhibitor cocktail and lysozyme. After 30 min, cells were sonicated 6 times for 30 sec with ultrasound (250 W). After centrifugation (20 000 x g, 10 min), the His-hFTO protein was purified using Ni^+^-Sepharose and size exclusion chromatography (Enrich SEC. 650). Purity of the protein was verified using SDS-PAGE. The purified protein was stored in the lysis buffer with 50% glycerol at 80 °C. Expression and purification of AlkB and ALKBH3 dioxygenases was performed as previously described^34,35^.

### Thermal shift assays for ligand binding

The assay was developed previously to monitor ligand binding to AlkB^36^. Fluorescence experiments were performed using a Varian Cary Eclipse spectrofluorimeter. Emission was monitored at 345 nm (excitation at 280 nm) over a temperature range of 40-95 °C with heating rate of 1 °C/min. The protein samples were dissolved in HEPES buffer at pH 7.5 with 2 mM dithiotreitol (DTT) and 150 mM NaCl to a final concentration of 1.2 µM. The process of thermal unfolding was performed at a concentration of 5 µM of the tested compound, either in the presence or absence of cofactors (50 µM 2OG and 100 µM Mn(II)). The thermal unfolding of the *holo* form of the protein was always monitored and further used as the reference. All the calculations were performed using Origin 9.0 software, assuming the simplest model of two-state (Folded-Unfolded) transition.

### *In vitro* inhibition assay

#### AlkB and ALKBH3

The reactions were carried out for 30 min at 37 °C in 20 µl of 50 mM Tris-HCl pH 7.5 with 1 mM DTT, 100 µM Fe(NH_4_)_2_(SO_4_)_2_, 50 µM 2OG, and 100 nM of AlkB or ALKBH3, 25 µM of substrate, and different concentrations of indicated inhibitors. Substrate contained 80% of TT(3mC)-TT and 20% TTCTT. 230 µl of ice cold water was added to stop the reaction and the mixtures were frozen to deactivate the enzyme^37^. Modified and unmodified (repaired) products were separated and analyzed on Knauer HPLC system with C18 column (Waters NovaPak). The mobile phase binding buffer (20 mM TEAA) and elution buffer (acetonitrile) were mixed and flown at a rate of 1 ml/min. The detection wavelength was 260 nm.

#### FTO

To measure the activity of obtained by us FTO protein, Chemiluminescent Assay Kit was used (BPS Bioscience). As a substrate, we used *N*^6^meA modification on RNA. The reactions were carried out according to the manufacturer’s protocol. Chemiluminescence was measured using the plate reader Synergy HT (Bio Tek) with 1 sec of integration time and 100 ms of delay after plate movement.

### Cell lines

In this work following cell lines were used:

1. EUFA30 – provided by Department of Toxicogenetis, Leiden University Medical Centre, The Netherlands; Passage numbers ~4;
2. HeLa – provided by Culture Collection, Public Health England, Porton Down, Salisbury, UK (Cat. No.: 93021013); Passage numbers ~4;
3. HEK-293 – provided by American Type Culture Collection, Manassas, USA (Cat. No.: ATCC-CRL-1573); Passage numbers ~4;
4. BICR18 – provided by Culture Collection, Public Health England, Porton Down, Salisbury, UK (Cat. No.: 06051601); Passage numbers ~4;
5. U-87 – provided by Culture Collection, Public Health England, Porton Down, Salisbury, UK (Cat. No.: 89081402); Passage numbers ~4;
6. JurkatE6-1 – provided by American Type Culture Collection, Manassas, USA (Cat. No.: ATCC® TIB-152™); Passage numbers ~4;
7. H929 –provided by American Type Culture Collection, Manassas, USA (Cat. No.: ATCC® CRL-9068™); Passage numbers ~4;

### Cell culture

HEK293, HeLa, U87, BICR 18, and EUFA30 cell lines were cultured in DMEM medium (Life Technology) supplemented with 10% fetal bovine serum (Life Technology) and 0.1% antibiotics (penicillin, streptomycin, Life Technology). Cells were grown in a humidified atmosphere of CO_2_/air (5/95%) at 37 °C.

### RNA interference of ALKBH1, 2, 3, 4, 5, 8 and FTO

Silencing was performed using the lipofectamine RNAiMAX reagent (Invitrogen) and ready-to-use siRNA mixture (Santa Cruz Biotechnology) composed of at least two different siRNAs targeting the same gene, to knock down *ALKBH1, 2, 3, 4, 5, 8,* and *FTO* (Supplementary Table 2). 1 × 10^6^ cells were seeded onto a 100 mm diameter dishes. After 24 h of incubation, siRNAs mixtures (with lipofectamine, in OPTI-MEM medium) were added. After 48 h, cells were collected for WB or flow cytometry analysis. For WB analysis, adherent cells were rinsed three times with PBS, scraped, and centrifuged (800 x g, 10 min, 4 °C). Next, cells were re-suspended in 100 μL RIPA buffer with a 1 x mammalian protease inhibitor cocktail (Sigma-Aldrich). The resulting lysates were spun down at 30 130 x g to remove insoluble particles, assayed for protein content using the Bradford assay, and loaded onto the SDS-PAGE at the protein amount of 20 μg per line. The strength of WB signals corresponding to the particular ALKBHs were calculated by densitometry. The expression changes by 30% were considered as significant.

### Viability assay

Exponentially growing cells at the density of 3 × 10^3^ cells/well were seeded onto a 96-well plate, cultured for 18 h followed by the respective treatment of antraquinones or its derivatives in various concentrations or 34 nM RNA interference of ALKBH proteins, and then cultured for a an additional 48 h. Alamar Blue (Invitrogen) viability assay was performed according to manufacturer’s protocol: after 4 h incubation, emission at 590 nm was measured with excitation at 560 nm using a scanning multiwall multimode spectrophotometer (DTX 880, Beckman Coulter). The experiment was carried out at least three times, with three replicates for each inhibitor concentration. After background subtraction, inhibition rates, IC50, values were calculated as the concentration of the component that inhibits cell growth by 50%. All the calculations were done using Origin 9.0 software.

### Flow cytometry

The Annexin V-FITC apoptosis detection kit (ApoAlert Annexin V-FITC Apoptosis Kit, TaKaRa) was used to detect apoptosis by flow cytometry. Cells were seeded at 6-well plates at concentration of 5 × 10^5^ cells/well, cultured for 18 h, and then the tested agent was applied for the indicated periods. Afterward, cells were washed with PBS, resuspended in binding buffer, and anti-Annexin V FITC-conjugated antibody and propidium iodide were added to 100 µl aliquots. The mixtures were incubated for 15 min at room temperature, supplemented with binding buffer to 500 µl, and processed using BD FACSCalibur (BD Biosciences). Data were analyzed in Flowing Software version 2.5.1 (Flowing Software, http://www.uskonaskel.fi/flowingsoftware).

### Molecular modelling

All calculations were performed with the aid of Yasara Structure package (ver. 17.1.28) using amber03 force-field. Initial structures of human ALKBH2 (3s57), ALKBH3 (2iuw), ALKBH5 (4oct), ALKBH7 (4qkd) ALKBH8 (3thp) and FTO (4zs2), rice ALKBH1 (5xeg) and *E*.*coli* (3i3q) were adopted from the appropriate PDB records. Each of these structures carried 2OG and metal cation (either Mn or Fe) in the catalytic centre, while all other ligands were removed. The structures of ALKBH1, ALKBH4 and ALKBH6 were modelled by homology on the basis PDB structures selected automatically by the combination of blast E-value, sequence coverage and structure quality. For each template, up to 5 alignments with the target sequence were used, and up to 50 different conformations were tested for each modelled loop. The resulting models were evaluated according to the structural quality (dihedral distribution, backbone and side-chain packing) and that with the highest score of these covering the largest part of the target sequence was used as the template for a hybrid model, which was further iteratively improved with the best fragments (e.g. loops) identified among the highly-scored single-template models.

Molecular Docking was done for each protein/ligand pair with the aid of VINA algorithm with 128 rounds of flexible docking applied against 16 replicas of protein structures (each representing re-optimized random ensemble of protein sidechain rotamers), and the resulting 2048 complexes were clustered with 2 Å threshold.

### Statistical analyses

All analyses were performed using R software (version 3.3.0, www.r-project.org) with *outliers* and *gplots* packages. The significance level α of 0.05 was assumed in all statistical tests. The Shapiro-Wilk test was used to assess agreement of ALKBHs content with the Gaussian distribution. Because even after filtering of extreme values with the Grubbs’ test for putative outliers^38^ the vast majority of the distributions were found to be non-Gaussian, further analyses were based on the non-parametric methods. The statistical significance of the differences observed in protein levels were tested using Wilcoxon tests; the paired (signed-rank) or non-paired versions (rank-sum, Mann-Whitney) were applied accordingly to the hypothesis being tested (i.e. the differences concerning either individuals or whole group analysed, respectively). Correlation matrixes were calculated for the levels of seven proteins using Spearman’s rank correlation coefficients. Hierarchical cluster analysis of these matrixes was performed according to the Ward criterion. To estimate the correlation between ALKBHs levels and G/TNM parameters, analysis of variance (ANOVA) and post-hoc pairwise t-student test with Benjamini Hochberg adjustment was used. In that case, samples were divided into groups according to G or TNM parameters. Distribution analysis for each group was also performed. Normal distribution of data sets of examined proteins was not be excluded (Shapiro-Wilk test)^39^.

### Ethics approval and consent to participate

All participants were informed about the purpose of the study and gave their written informed consent. The study was approved by Warsaw Medical University Bioethical Committee permission for working with human tissues/tumours no. KBO/17/11 April 12th 2011. All research was performed in accordance with the relevant guidelines and regulations.

## Supplementary information

Supplementary info

## Data Availability

Please contact the corresponding author for all data requests.
