# Supplementary material for: ALKBH overexpression in head and neck cancer: potential target for novel anticancer therapy
Source: Sci Rep. 2019 Sep 13;9:13249. doi: 10.1038/s41598-019-49550-x (PMC6744417; doi:10.1038/s41598-019-49550-x)
Supplement: Supplementary file 1 — Supplementary info [file 41598_2019_49550_MOESM1_ESM.docx]

**Supplement**

**ALKBH overexpression in head and neck cancer: potential target for novel anticancer therapy**

Tomaš Pilžys^1#^, Michał Marcinkowski^1#^, Wojciech Kukwa^2#^, Damian Garbicz^1^, Małgorzata Dylewska^1^, Karolina Ferenc^3^, Adam Mieczkowski^1^, Andrzej Kukwa^2^, Ewa Migacz^2^, Dominika Wołosz^4^, Damian Mielecki^1^, Arne Klungland^5^, Jan Piwowarski^1^, Jarosław Poznański^1^*, Elżbieta Grzesiuk^1^*

^1^ Institute of Biochemistry and Biophysics, Polish Academy of Sciences, Warsaw, Poland

^2^ Department of Otolaryngology, Medical University of Warsaw, Poland

^3^ Veterinary Research Centre and Center for Biomedical Research, Department of Large Animal Diseases with the Clinic, Faculty of Veterinary Medicine, Warsaw University of Life Sciences

^4^ Department of Pathology, Medical University of Warsaw, Poland

^5^ Department of Microbiology, Oslo University Hospital, Oslo, Norway


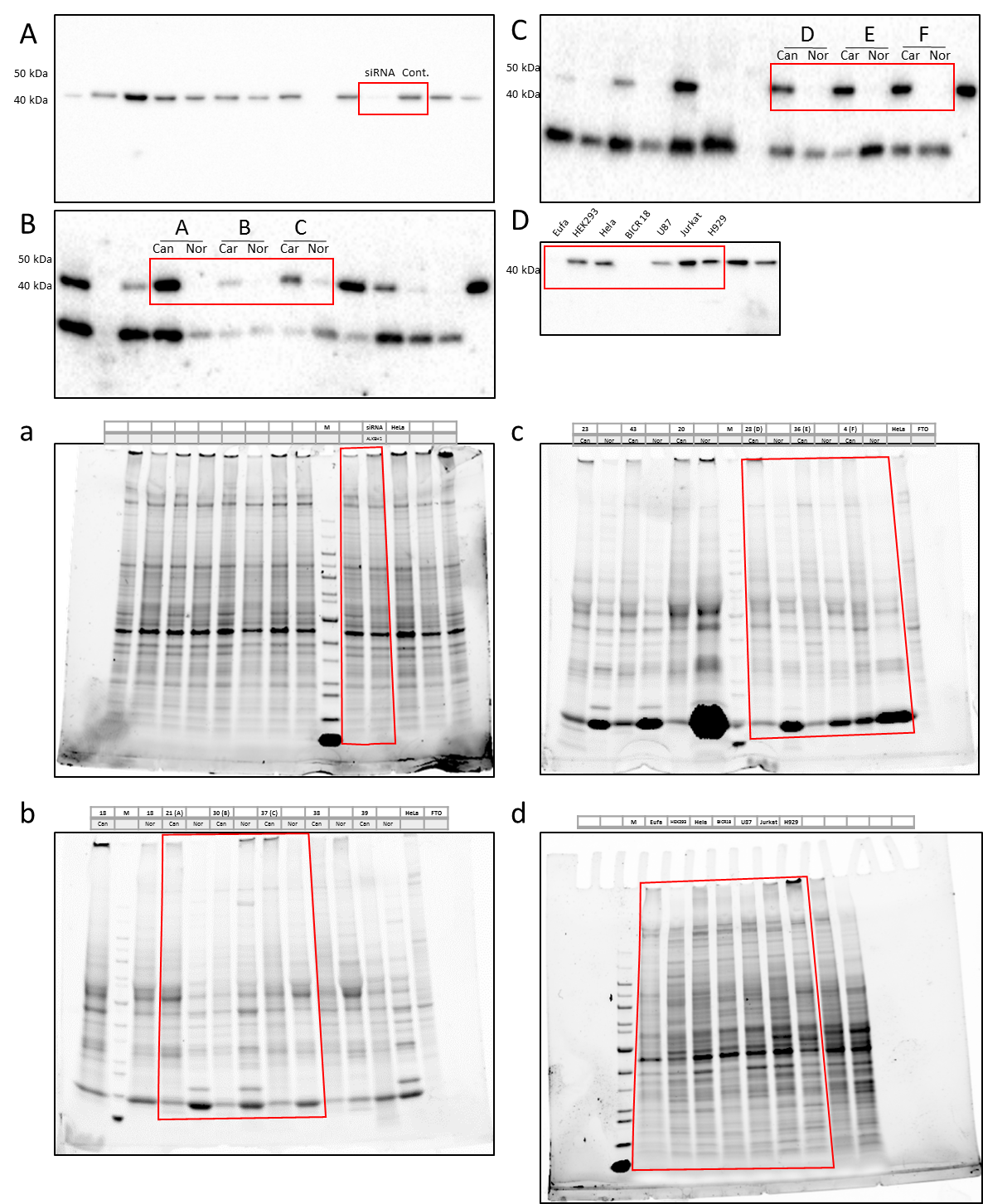


**Figure S1. Western blot analysis of ALKBH1 protein.** (A) RNA interference verification. siRNA - HeLa cells treated with siRNA of ALKBH1; Cont. - HeLa cells not treated by siRNA. (B, C) ALKBH1 expression in HNSCC. Nor - normal periphery; Can- cancer; A-F - tumour samples. (D) ALKBH1 expression in various cell lines. (a, b, c, d) – Stain Free gels representing loading controls for corresponding blots.


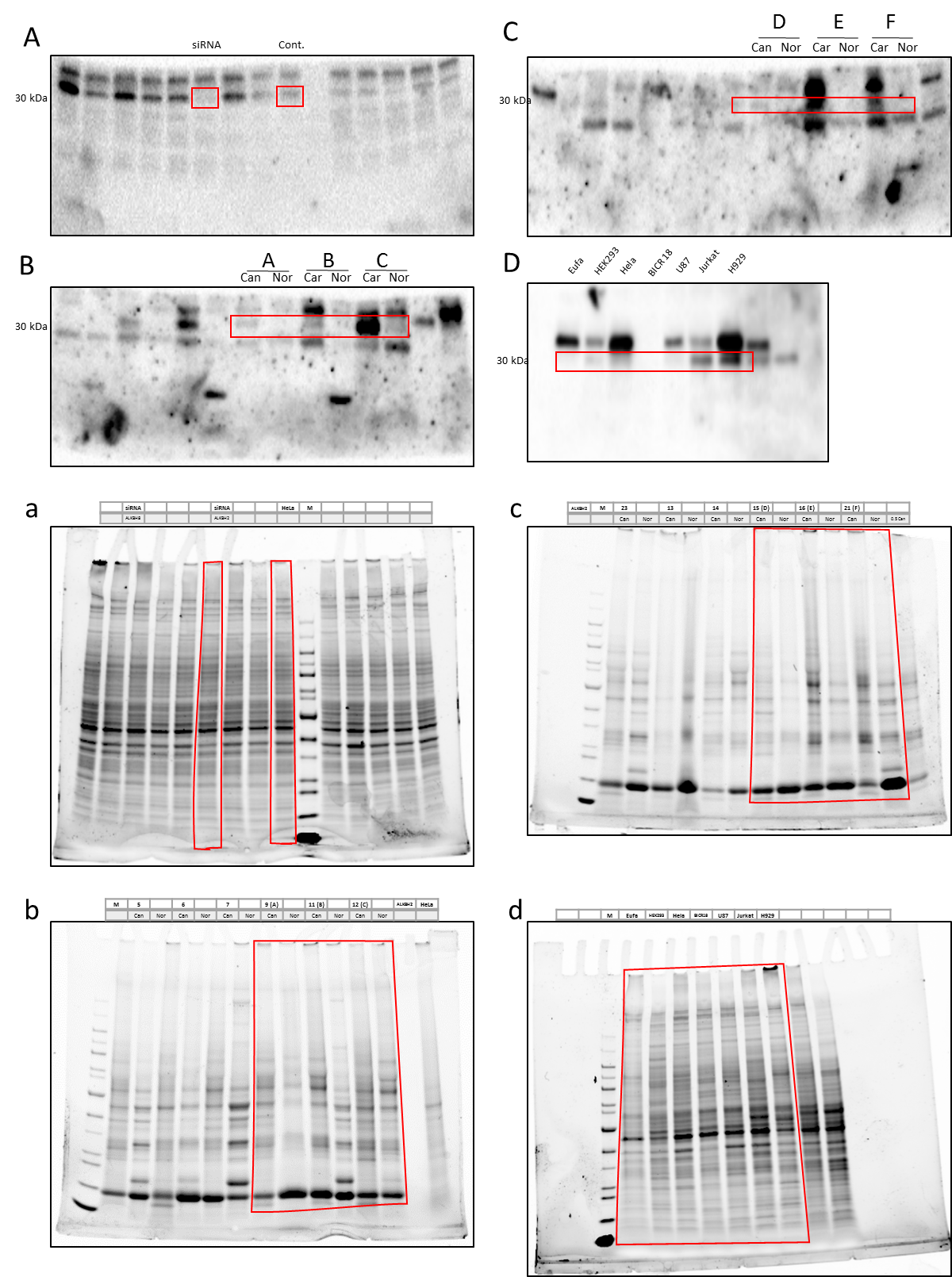


**Figure S2. Western blot analysis of ALKBH2 protein.** (A) RNA interference verification. siRNA - HeLa cells treated with siRNA of ALKBH2; Cont. - HeLa cells not treated by siRNA. (B, C) ALKBH2 expression in HNSCC. Nor - normal periphery; Can- cancer; A-F - tumour samples. (D) ALKBH2 expression in various cell lines. (a, b, c, d) – Stain Free gels representing loading controls for corresponding blots.


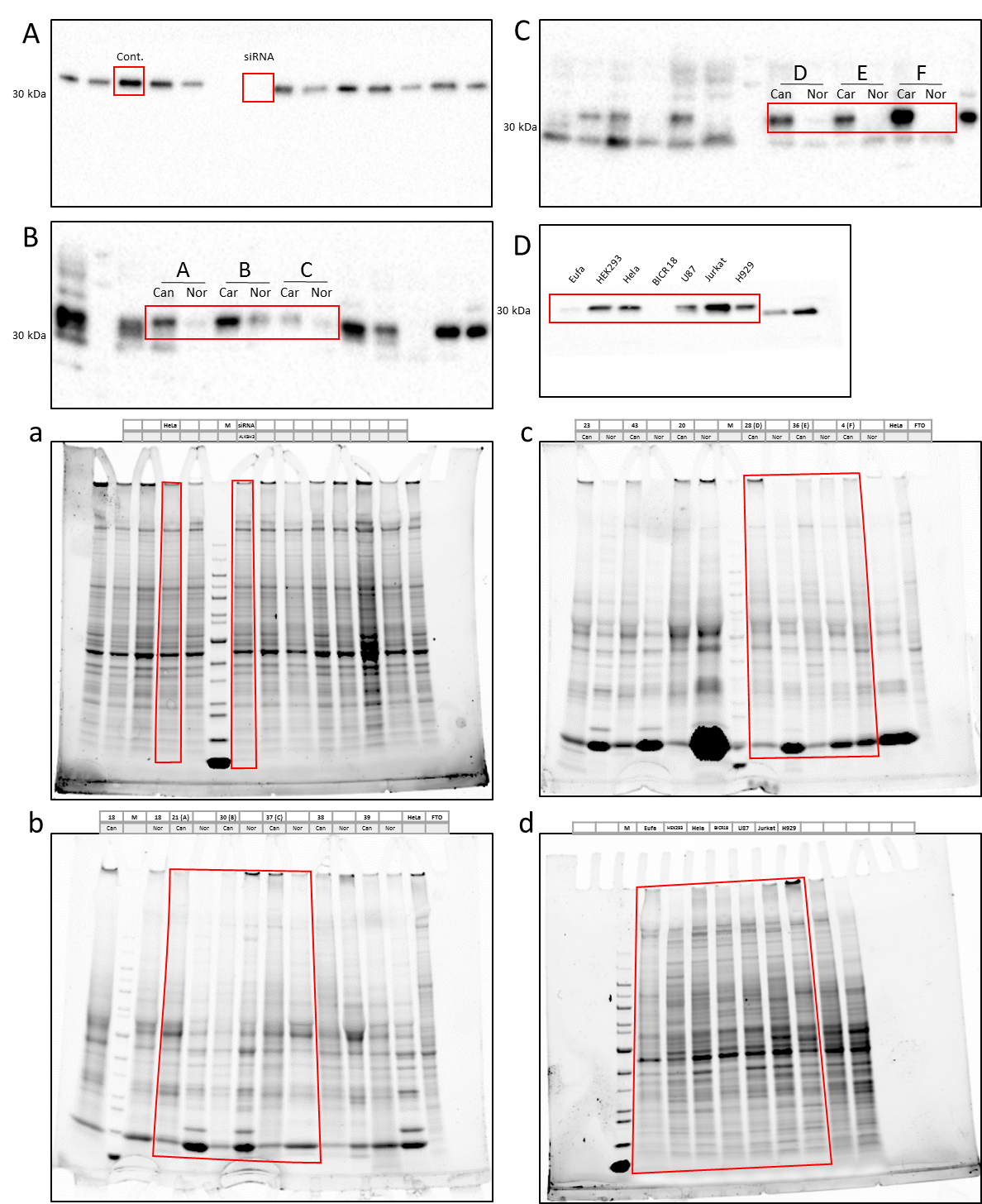


**Figure S3. Western blot analysis of ALKBH3 protein.** (A) RNA interference verification. siRNA - HeLa cells treated with siRNA of ALKBH3; Cont. - HeLa cells not treated by siRNA. (B, C) ALKBH3 expression in HNSCC. Nor - normal periphery; Can- cancer; A-F - tumour samples. (D) ALKBH3 expression in various cell lines. (a, b, c, d) – Stain Free gels representing loading controls for corresponding blots.


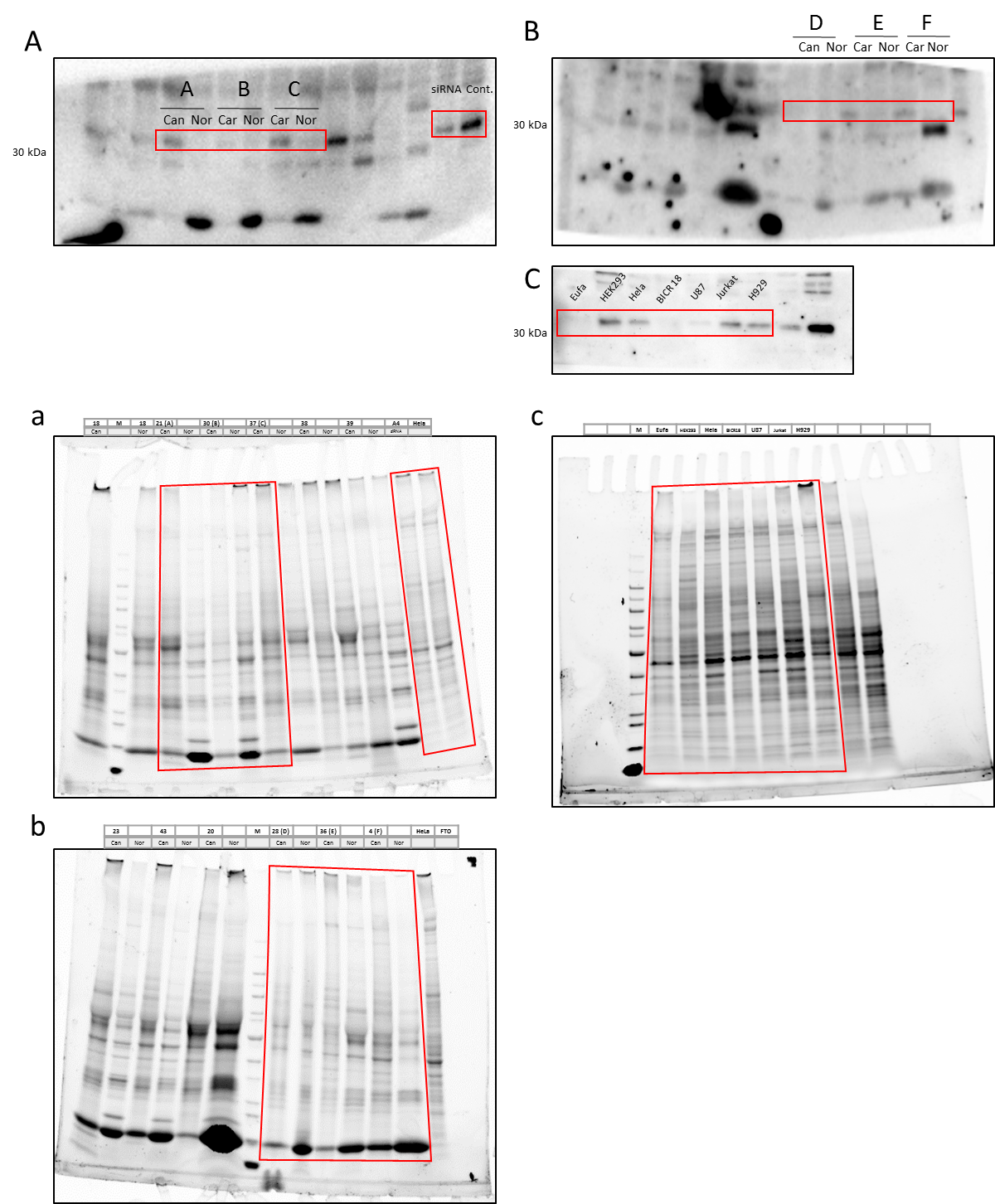


**Figure S4.** **Western blot analysis of ALKBH4 protein.** (A) RNA interference verification. siRNA - HeLa cells treated with siRNA of ALKBH4; Cont. - HeLa cells not treated by siRNA. (A, B) ALKBH4 expression in HNSCC. Nor - normal periphery; Can- cancer; A-F - tumour samples. (C) ALKBH4 expression in various cell lines. (a, b, c) – Stain Free gels representing loading controls for corresponding blots.


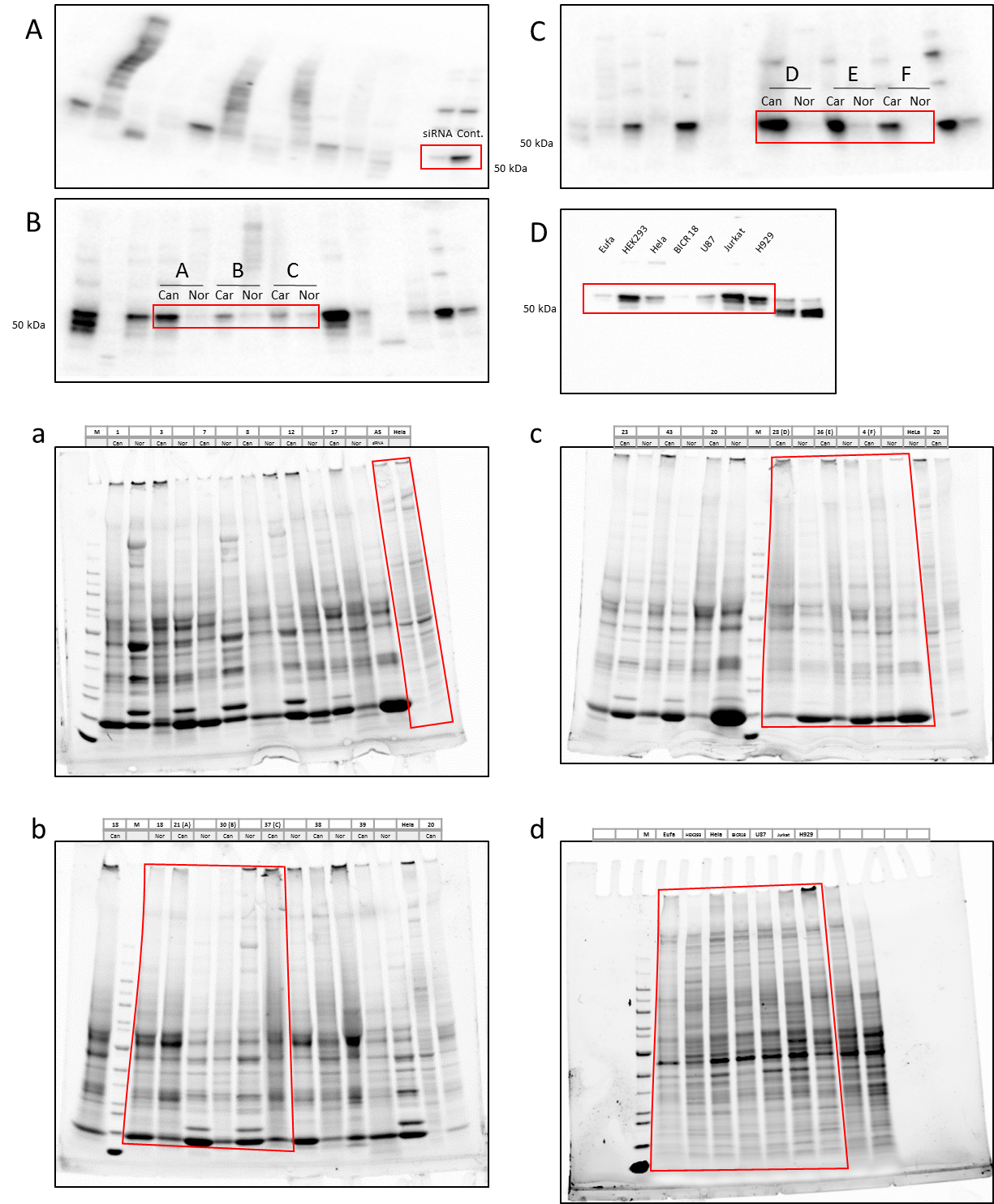


**Figure S5. Western blot analysis of ALKBH5 protein.** (A) RNA interference verification. siRNA - HeLa cells treated with siRNA of ALKBH5; Cont. - HeLa cells not treated by siRNA. (A, B) ALKBH5 expression in HNSCC. Nor - normal periphery; Can- cancer; A-F - tumour samples. (C) ALKBH5 expression in various cell lines. (a, b, c, d) – Stain Free gels representing loading controls for corresponding blots.


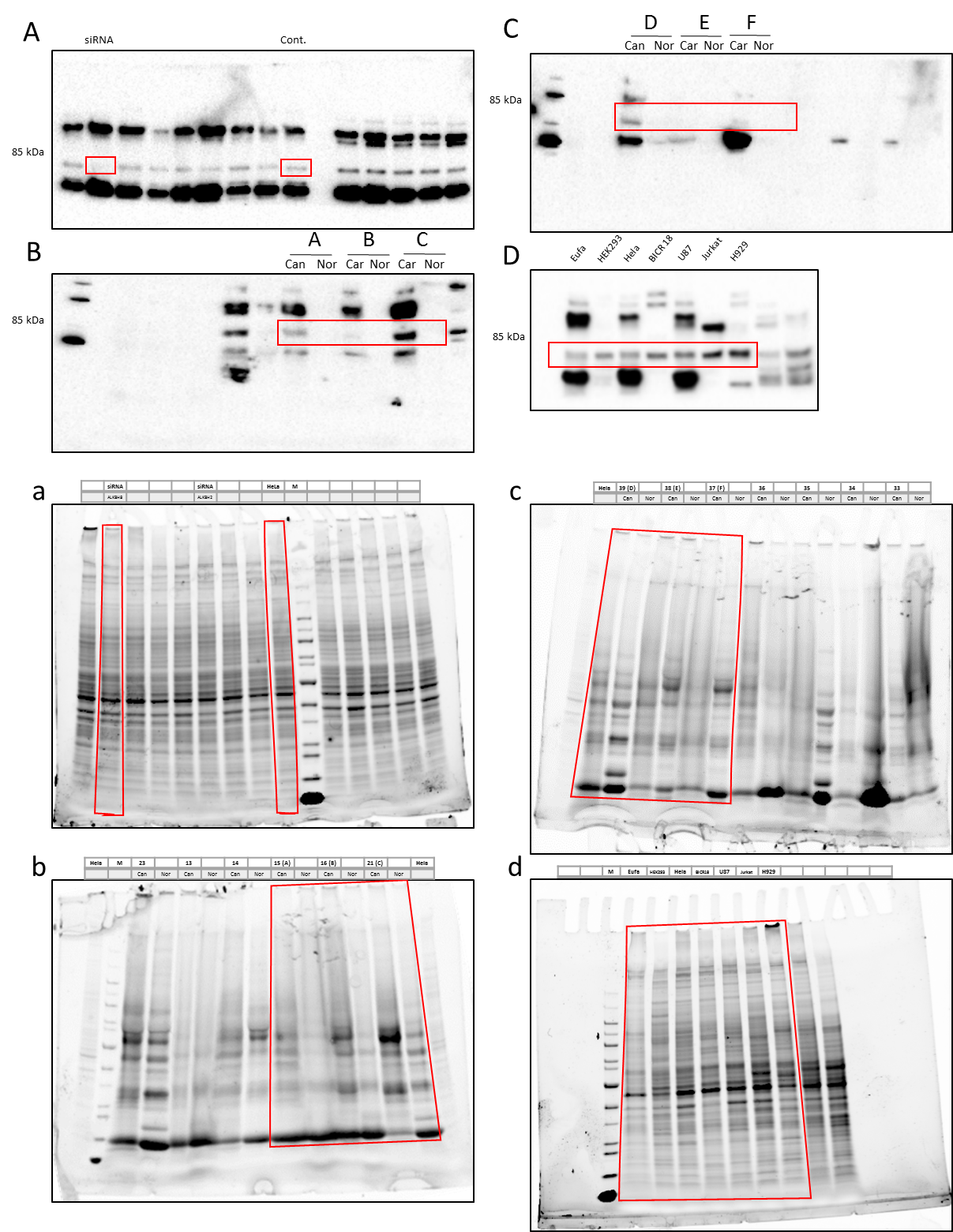


**Figure S6. Western blot analysis of ALKBH8 protein.** (A) RNA interference verification. siRNA - HeLa cells treated with siRNA of ALKBH8; Cont. - HeLa cells not treated by siRNA. (B, C) ALKBH8 expression in HNSCC. Nor - normal periphery; Can- cancer; A-F - tumour samples. (D) ALKBH8 expression in various cell lines. (a, b, c, d) – Stain Free gels representing loading controls for corresponding blots.


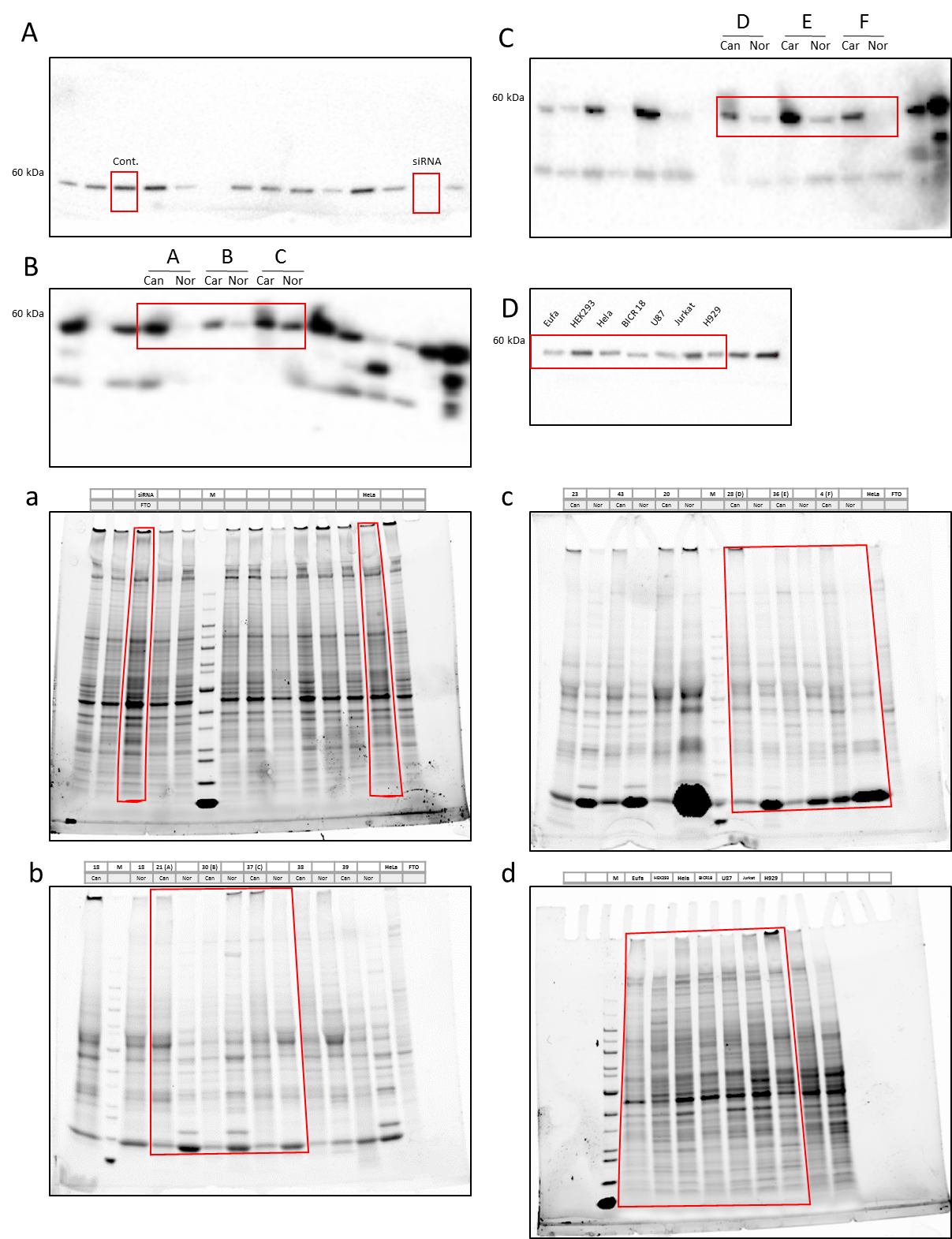


**Figure S7. Western blot analysis of FTO protein.** (A) RNA interference verification. siRNA - HeLa cells treated with siRNA of FTO; Cont. - HeLa cells not treated by siRNA. (B, C) FTO expression in HNSCC. Nor - normal periphery; Can- cancer; A-F - tumour samples. (D) FTO expression in various cell lines. (a, b, c, d) – Stain Free gels representing loading controls for corresponding blots.

**
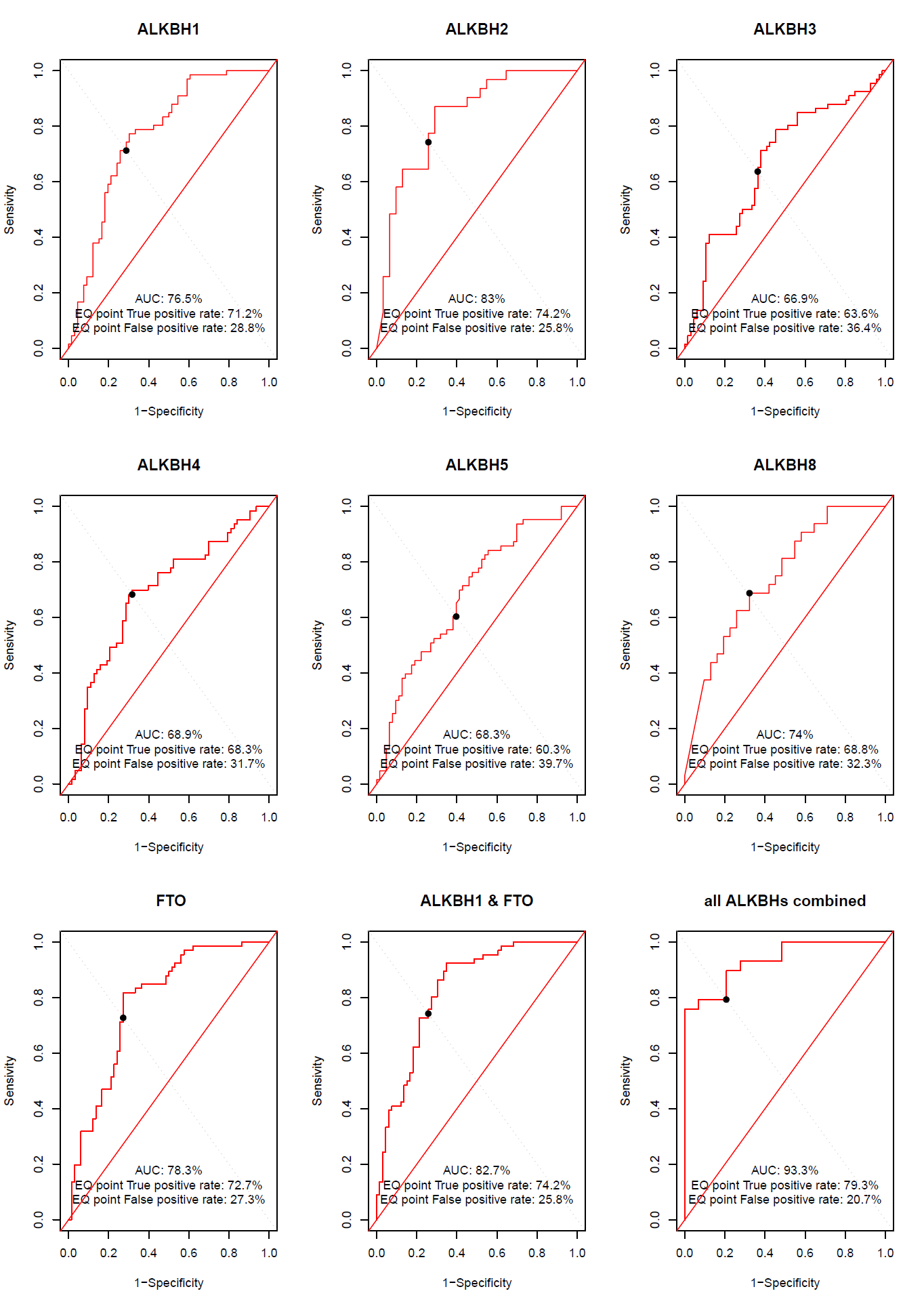
**

**Figure S8. ROC curves comparison for cancer and adjacent tissues of HNSCC patients according to the level of each ALKBH protein and ALKBHs combined.**

**
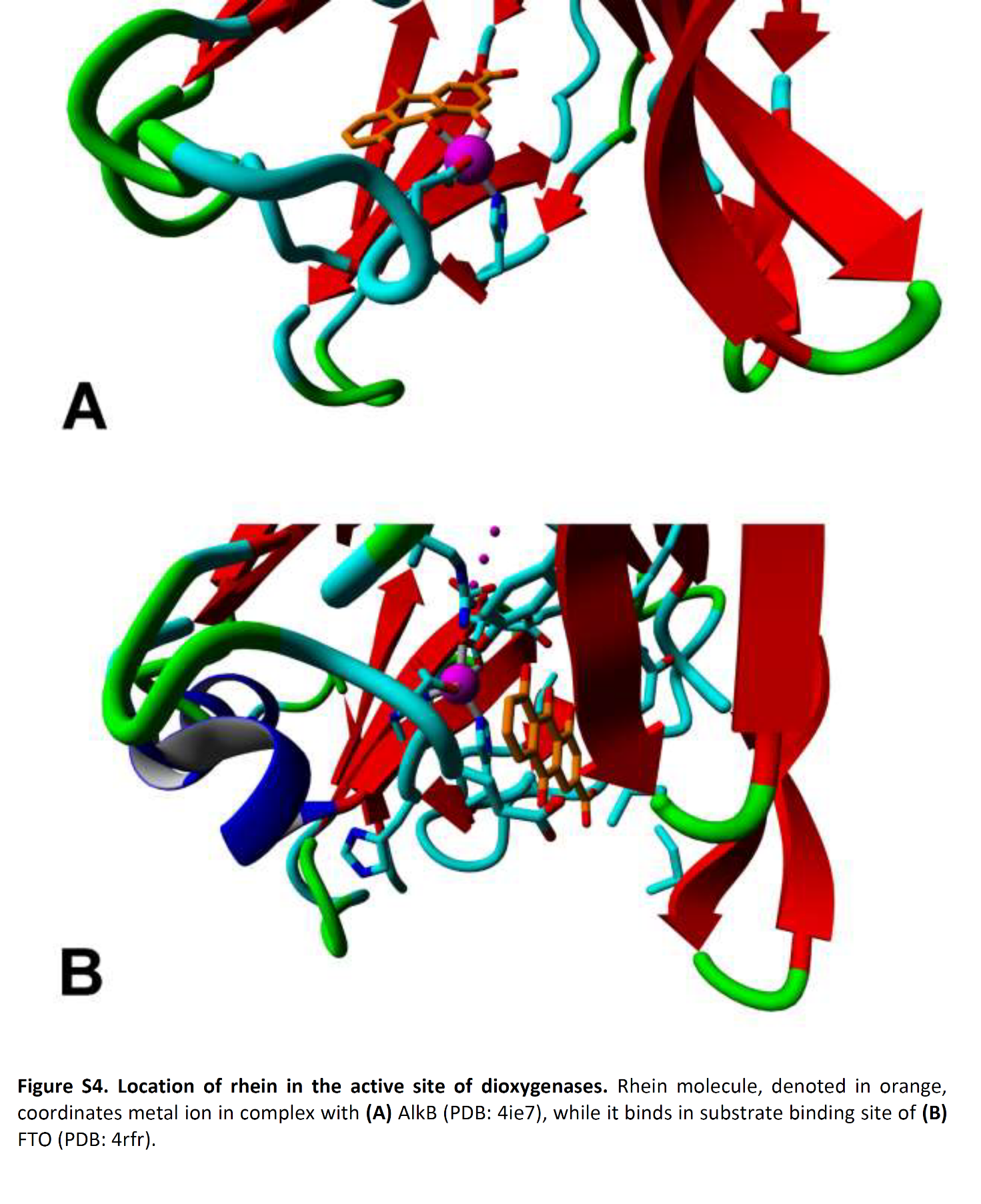
**

**Figure S9. Location of rhein in the active site of dioxygenases.** Rhein molecule, denoted in orange, coordinates metal ion in complex with **(A)** AlkB (PDB: 4rfr) while it binds in substrate binding site of **(B)** FTO (PDB: 4ie7).


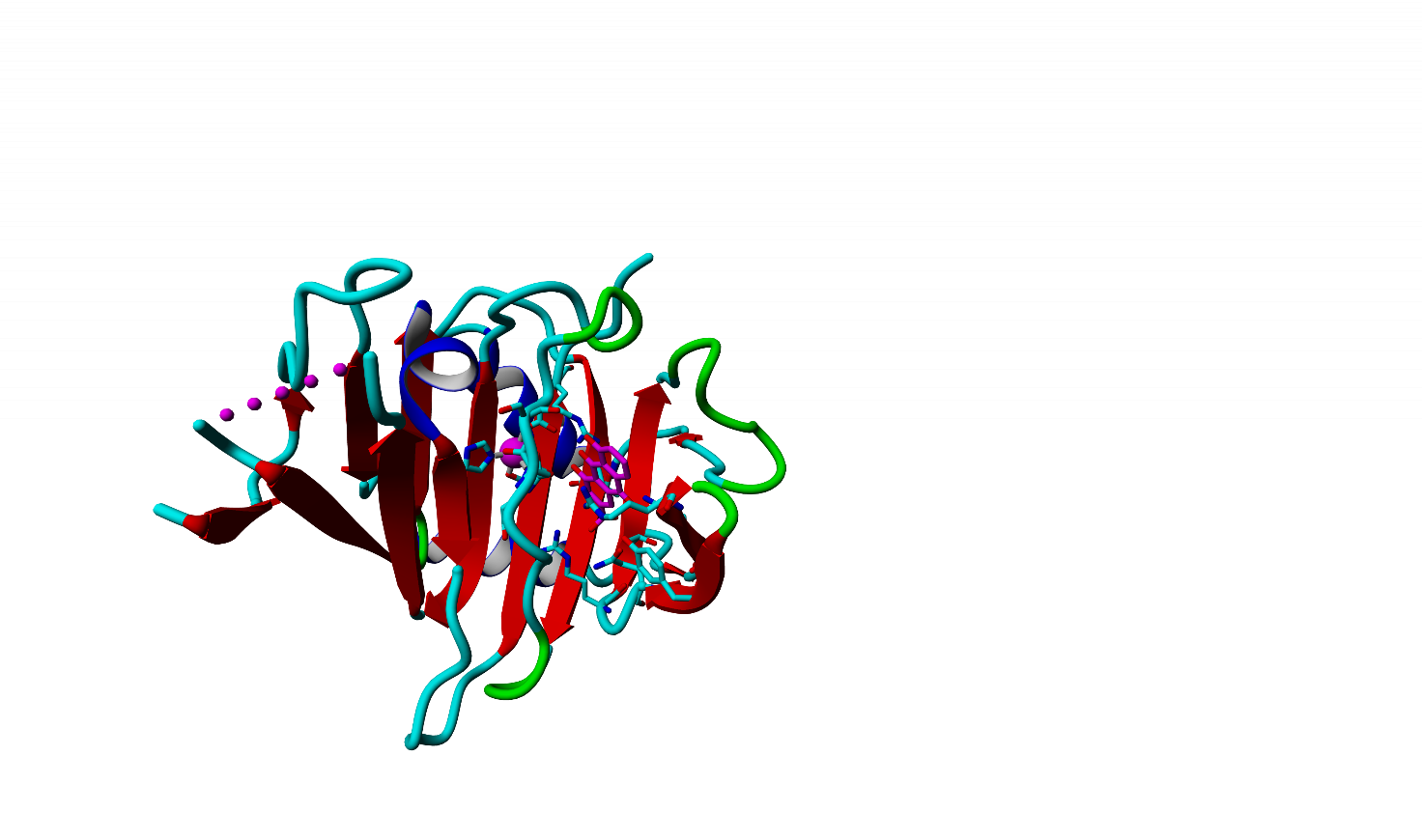


**Figure S10. Representative structure of ALKBH3 – rhein complex.**

**ALKBH3**


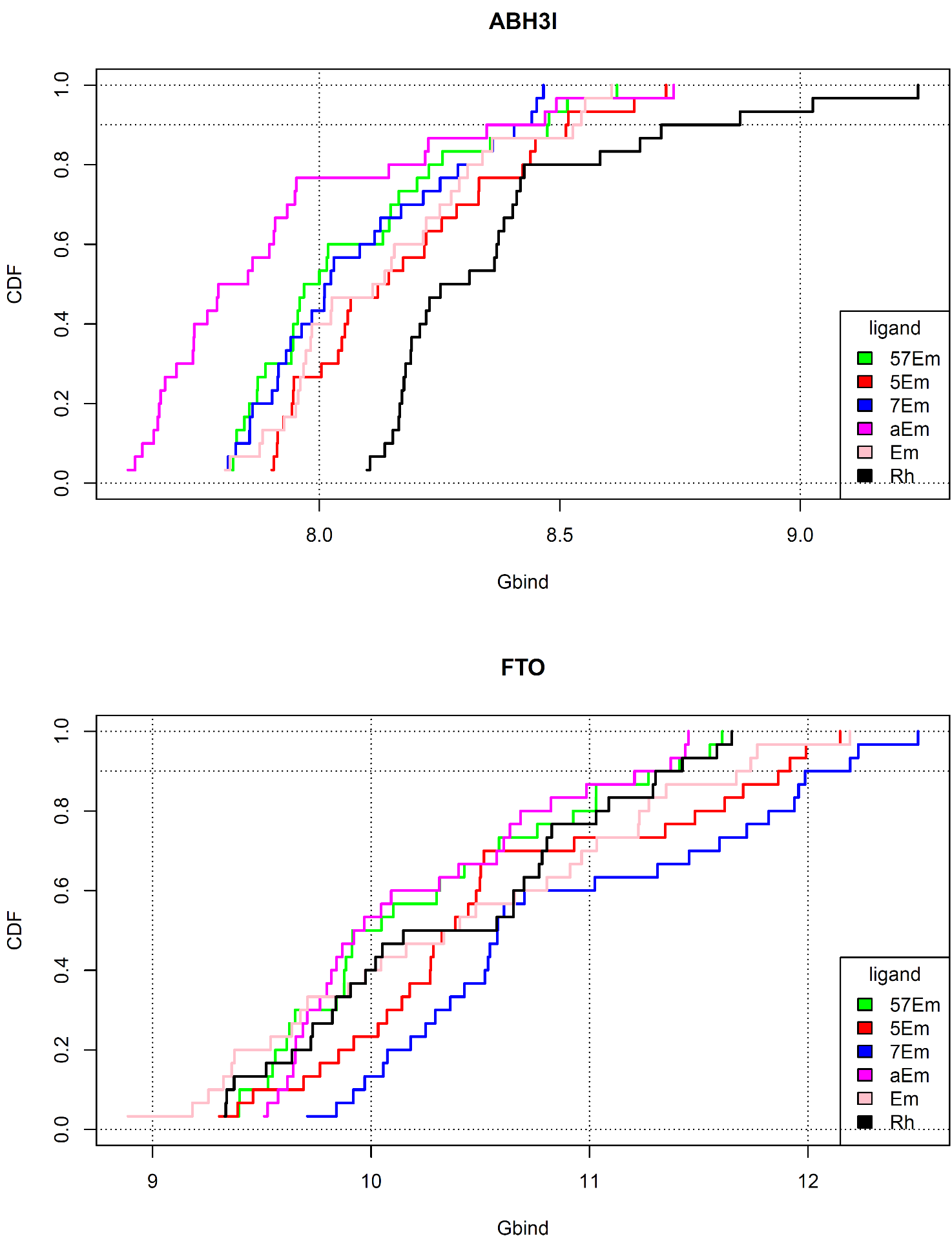

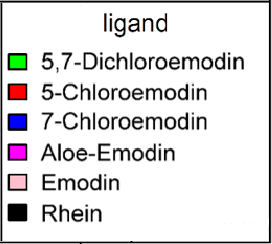

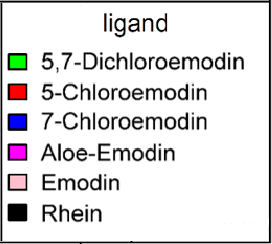


**Figure S11. *In silico* analysis for the binding of antraquinone derivatives by ALKBH3 and FTO**

**
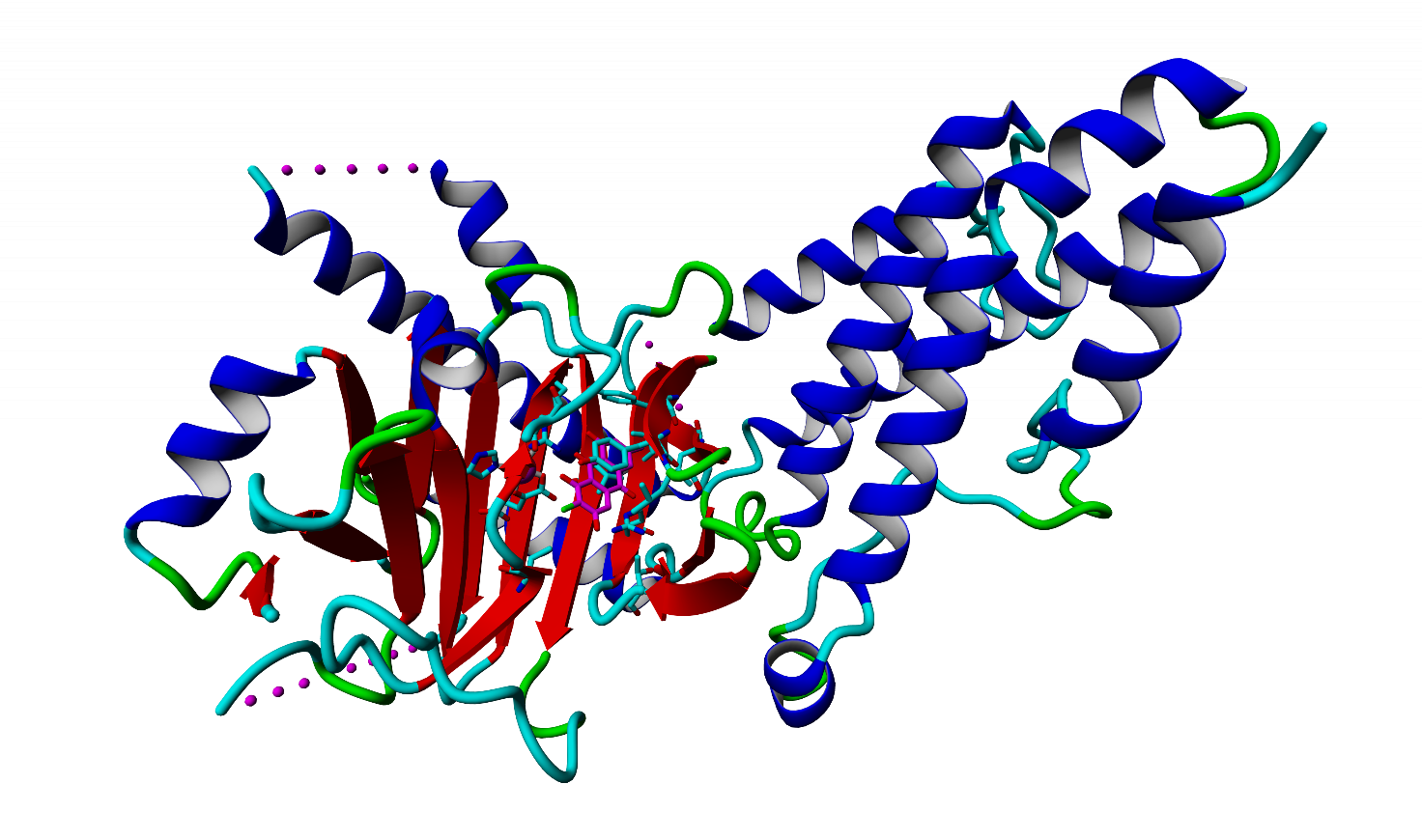
**

**Figure S12. Representative structure of FTO – 7-chloroemodin complex.**

**Figure S13.** **Structures of anthraquinone derivatives used in this study.** (1) Emodin, (2) Aloe-Emodin, (3) Rhein, (4) 7-Chloroemodin, (5) 5-Chloroemodin, (6) 5,7-Dichloroemodin, (7) Carminic acid.

**
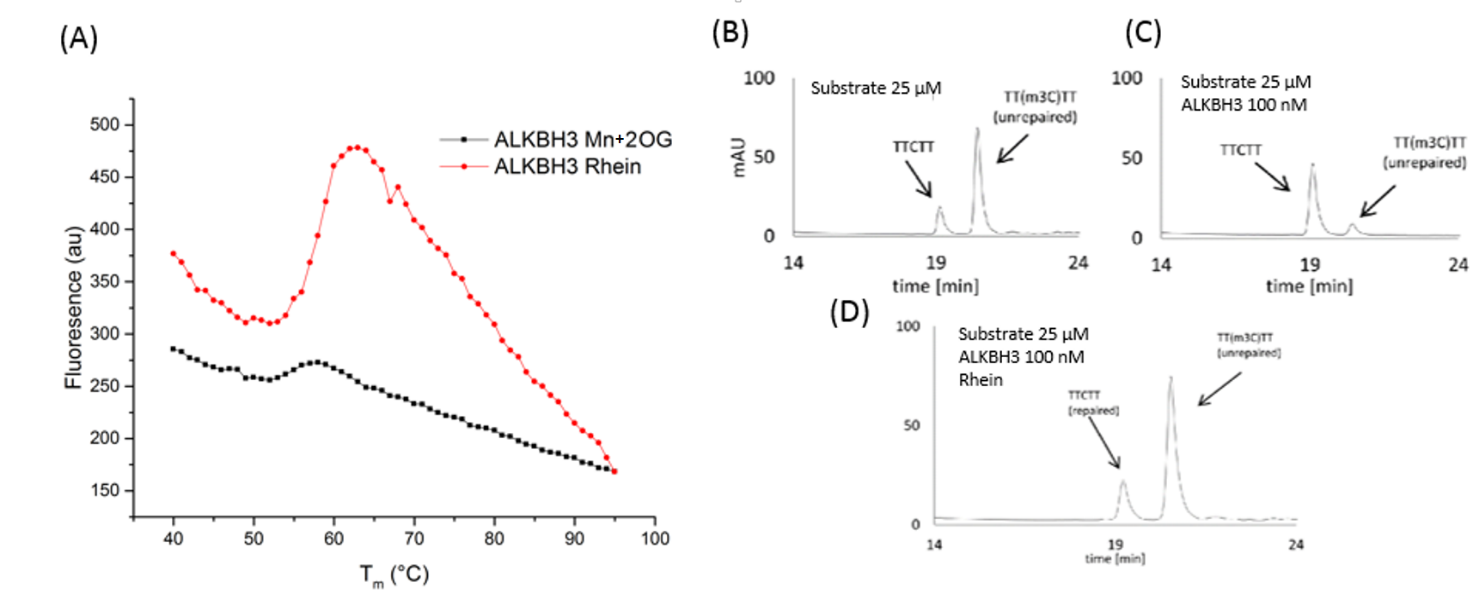
**

**Figure S14. Rhein’s influence on ALKBH3 stability and activity.** (A) Melting profile of ALKBH3 (B),(C),(D) Results of HPLC samples after enzymatic demethylation of 3meC done by ALKBH3.


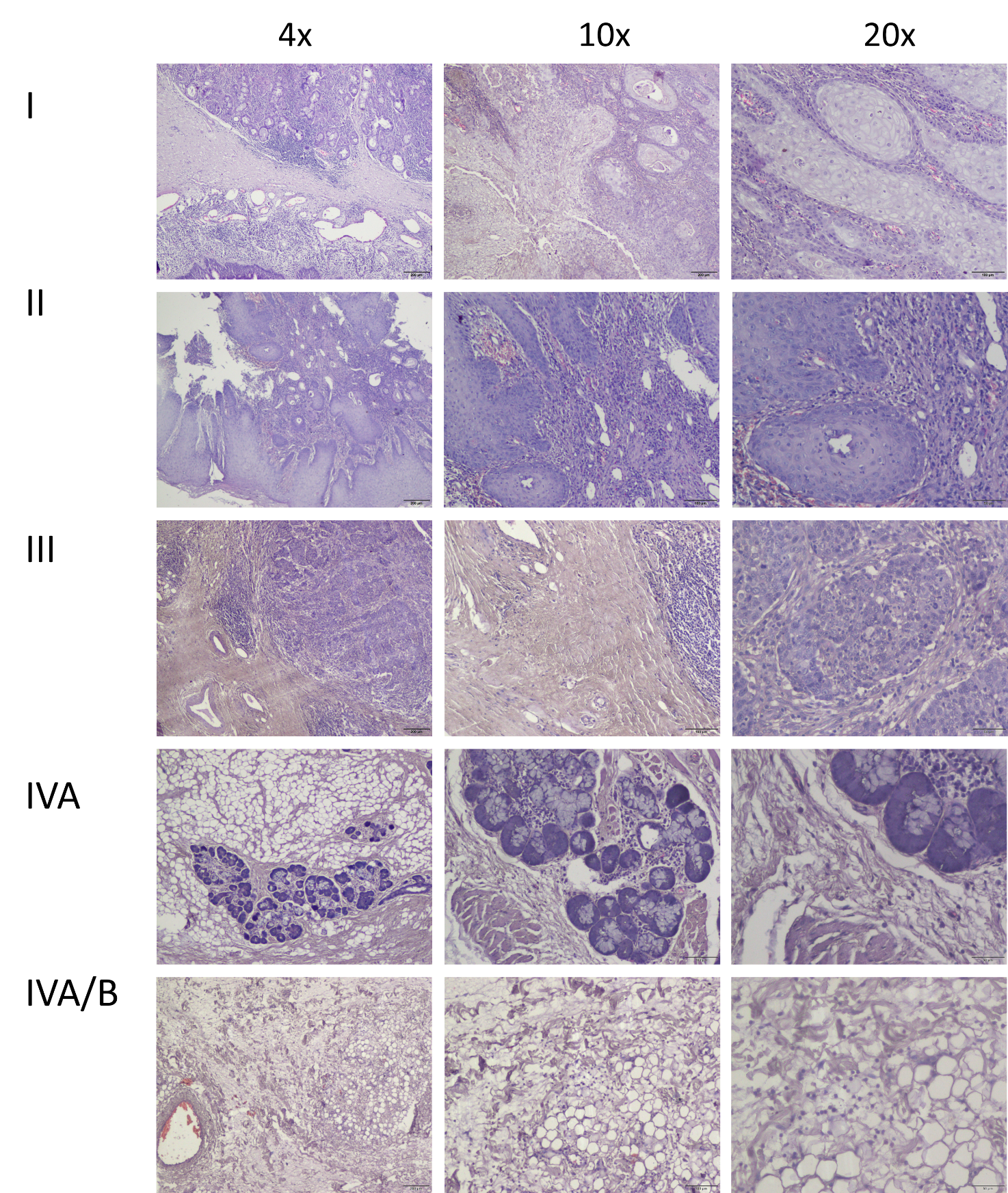


**Figure S15. Hematoxilin- Eosin staining of HNSCC tissues.** Histopatology evaluation of cancer stage (form I to IV) using TMN scale. Objective: 4x, 10x and 20x.

**Table SI. The properties of human ALKBH dioxygenases.**

| Protein | Localization | Substrates/modification | Protein Function | Citation |
| --- | --- | --- | --- | --- |
| AlkB  24.1kDa | Cytoplasm | DNA and RNA (1-methyladenine, 3‑methylcytosine)  DNA (1-ethyladenine, 1‑propyladenine, ethenoadenine, others) | DNA, RNA repair enzyme | ^1,2^ |
| ALKBH1  43.8 kDa | Nucleus, mitochondrium | Protein (methyl-Lysine),  DNA (N6-methyladenine, 3‑methylcytosine)  RNA (3-methylcytosine),  tRNA (N1-methyladenine, 5‑methylcytosine) | Histone demethylase  Acts as a regulator of translation initiation and elongation in response to glucose deprivation.  DNA lyase activity, introduces double-stranded breaks at abasic sites, cleaves ssDNA and dsDNA at abasic site.  Deletion influences early mammalian development e.g. intra-uterine growth retardation or placental defect. | ^3,4^ |
| ALKBH2  29.3 kDa | Nucleus | DNA (1-methyladenine, 3‑methylcytosine, 1‑ethenoadenine  prefers dsDNA structures, | Interacts with the proliferating cell nuclear antigen (PCNA), creating ALKBH2-PCNA-interating motif (APIM)  Deletions are viable with no obvious phenotype in mouse | ^5,6^ |
| ALKBH3  33.4 kDa | Nucleus, cytoplasm | DNA/RNA (1-methyladenosine, 3‑methylcytosine), prefers ssDNA structures, | Associates with the activating signal cointegrator complex (ASCC)  In mouse, deletions are viable with no obvious phenotype | ^3,7–9^ |
| ALKBH4  33.8 kDa | Nucleus, cytoplasm | Protein (methyl-Lysine) | Mediates demethylation of actin monomethylated lysine (K84me1);  Required for recruitment of NM II to actin;  Deletion leads to impaired spermatogenesis and is lethal in early embryonic development | ^8,10^ |
| ALKBH5  52.3 kDa | Nucleus, nucleus speckle | DNA and RNA (N6-methyladenosine) | Affects mRNA export and RNA metabolism;  Regulatory role under conditions of inadequate cellular oxygenation;  Deletion influences mouse fertility, aberrant spermatogenesis, and oogenesis | ^7^ |
| ALKBH6  26.5 kDa | Nucleus, cytoplasm | Unknown | Unknown | ^8^ |
| ALKBH7  24.5 kDa | Mitochondrial matrix | Protein (*in vitro* catalyzes auto-hydroxylation at Leu-110) does not display DNA demethylase activity | Involved in fatty acid metabolism;  Required for programmed necrosis after oxidative stress;  Deletion are viable and lead to increased body weight and fat in mouse | ^7^ |
| ALKBH8  75.2 kDa | Nucleus, cytoplasm | tRNA (5-carboxymethyl uridine) | Methyltransferase, required for the final step in the biogenesis of 5-methoxycarbonylmethyluridine (mcm5u) in tRNAs  Deletion are viable but might be disadvantageous under conditions of increased oxidative stress | ^11,12^ |
| ALKBH9 (FTO)  58.3 kDa | Nucleus,  Nucleus speckle | RNA (N6-methyladenosine, 3‑methyluracil, 3-methylthymine),  DNA (3-methylthymine) has no detectable activity towards dsDNA | Affects mRNA metabolism;  Contributes to the regulation of body size and body fat accumulation;  Involved in the regulation of thermogenesis and the control of adipocyte differentiation into brown or white fat cells;  Deletion leads to growth retardation, loss of white adipose tissue or multiple malformations | ^13,14^ |

**Table SII. siRNA mixuture used for silecing of *ALKBH* genes**

| Target | Sequence |
| --- | --- |
| ALKBH1 | sc-60153A:  • Sense: GCAAGCCUAUGGACUCAAAtt  • Antisense: UUUGAGUCCAUAGGCUUGCtt  sc-60153B:  • Sense: GGGAAGAACUCAUUGUUGAtt  • Antisense: UCAACAAUGAGUUCUUCCCtt  sc-60153C:  • Sense: GCUGUAUUGUUCAUGCUAUtt  • Antisense: AUAGCAUGAACAAUACAGCtt |
| ALKBH2 | sc-96063A:  • Sense: CCUUCAACUUUGUGCUCAUtt  • Antisense: AUGAGCACAAAGUUGAAGGtt  sc-96063B:  • Sense: GUCUUCCGGCAUAAGGAUUtt  • Antisense: AAUCCUUAUGCCGGAAGACtt |
| ALKBH3 | sc-96711A:  • Sense: GUAUCUAGGGUCUGUUUGUtt  • Antisense: ACAAACAGACCCUAGAUACtt  sc-96711B:  • Sense: GAGAACUUCCUUACACUUAtt  • Antisense: UAAGUGUAAGGAAGUUCUCtt  sc-96711C:  • Sense: CAUGGGACCUUGUUAAUCAtt  • Antisense: UGAUUAACAAGGUCCCAUGtt |
| ALKBH4 | sc-89850A:  • Sense: CGGAAACAGAAGCUAAAGAtt  • Antisense: UCUUUAGCUUCUGUUUCCGtt  sc-89850B:  • Sense: CCCAUAACGCUGUUAACAAtt  • Antisense: UUGUUAACAGCGUUAUGGGtt  sc-89850C:  • Sense: GGCUUUACCACCAUGUCAAtt  • Antisense: UUGACAUGGUGGUAAAGCCtt |
| ALKBH5 | sc-93856A:  • Sense: CAGUGGAUAUGCUGCUGAUtt  • Antisense: AUCAGCAGCAUAUCCACUGtt  sc-93856B:  • Sense: CUGUUAGGGCUGAAGAAUAtt  • Antisense: UAUUCUUCAGCCCUAACAGtt  sc-93856C:  • Sense: CAUCCCAAAGCUAUUGUUAtt  • Antisense: UAACAAUAGCUUUGGGAUGtt |
| ALKBH8 | sc-96576A:  • Sense: GCAUUGCAGUGCCAGUUAUtt  • Antisense: AUAACUGGCACUGCAAUGCtt  sc-96576B:  • Sense: CAGUGAUGUUGGAGACUUAtt  • Antisense: UAAGUCUCCAACAUCACUGtt  sc-96576C:  • Sense: GGAAGGCACUCAUUUAUGUtt  • Antisense: ACAUAAAUGAGUGCCUUCCtt |
| FTO | sc-75002A:  • Sense: CCAUAAAGAGGUUCAAGAAtt  • Antisense: UUCUUGAACCUCUUUAUGGtt  sc-75002B:  • Sense: CUACAACGGACAAGAUGAAtt  • Antisense: UUCAUCUUGUCCGUUGUAGtt  sc-75002C:  • Sense: GGAUGACUCUCAUCUCGAAtt  • Antisense: UUCGAGAUGAGAGUCAUCCtt |

**References**

1. Falnes, P. Ø., Johansen, R. F. & Seeberg, E. AlkB-mediated oxidative demethylation reverses DNA damage in Escherichia coli. *Nature* **419,** 178–182 (2002).

2. Aas, P. A. *et al.* Human and bacterial oxidative demethylases repair alkylation damage in both RNA and DNA. *Nature* **421,** 859–863 (2003).

3. Li, Q. *et al.* Rhein inhibits AlkB repair enzymes and sensitizes cells to methylated DNA damage. *J. Biol. Chem.* **291,** 11083–11093 (2016).

4. Haag, S. *et al.* NSUN3 and ABH1 modify the wobble position of mt‐tRNA ^Met^ to expand codon recognition in mitochondrial translation. *EMBO J.* **35,** 2104–2119 (2016).

5. Ringvoll, J. *et al.* AlkB homologue 2-mediated repair of ethenoadenine lesions in mammalian DNA. *Cancer Res.* **68,** 4142–4149 (2008).

6. Gilljam, K. M. *et al.* Identification of a novel, widespread, and functionally important PCNA-binding motif. *J. Cell Biol.* **186,** 645–654 (2009).

7. Feng, C. *et al.* Crystal structures of the human RNA demethylase alkbh5 reveal basis for substrate recognition. *J. Biol. Chem.* **289,** 11571–11583 (2014).

8. Tsujikawa, K. *et al.* Expression and sub-cellular localization of human ABH family molecules. *J. Cell. Mol. Med.* **11,** 1105–1116 (2007).

9. Dango, S. *et al.* DNA unwinding by ASCC3 helicase is coupled to ALKBH3-dependent DNA alkylation repair and cancer cell proliferation. *Mol. Cell* **44,** 373–384 (2011).

10. Li, M. M. *et al.* ALKBH4-dependent demethylation of actin regulates actomyosin dynamics. *Nat. Commun.* **4,** (2013).

11. Songe-Moller, L. *et al.* Mammalian ALKBH8 Possesses tRNA Methyltransferase Activity Required for the Biogenesis of Multiple Wobble Uridine Modifications Implicated in Translational Decoding. *Mol. Cell. Biol.* **30,** 1814–1827 (2010).

12. Fu, D. *et al.* Human AlkB Homolog ABH8 Is a tRNA Methyltransferase Required for Wobble Uridine Modification and DNA Damage Survival. *Mol. Cell. Biol.* **30,** 2449–2459 (2010).

13. Zhou, J. *et al.* Dynamic m6A mRNA methylation directs translational control of heat shock response. *Nature* **526,** 591–594 (2015).

14. Claussnitzer, M. *et al.* *FTO* Obesity Variant Circuitry and Adipocyte Browning in Humans. *N. Engl. J. Med.* **373,** 895–907 (2015).
